# Multifaceted Roles of Metzincins in CNS Physiology and Pathology: From Synaptic Plasticity and Cognition to Neurodegenerative Disorders

**DOI:** 10.3389/fncel.2017.00178

**Published:** 2017-06-30

**Authors:** Patrycja Brzdak, Daria Nowak, Grzegorz Wiera, Jerzy W. Mozrzymas

**Affiliations:** ^1^Department of Physiology and Molecular Neurobiology, Wroclaw UniversityWroclaw, Poland; ^2^Laboratory of Neuroscience, Department of Biophysics, Wroclaw Medical UniversityWroclaw, Poland

**Keywords:** metzincins, metalloproteinases, synaptic plasticity, learning, amyloid β, Alzheimer’s disease, blood-brain barrier, blood-cerebrospinal fluid barrier

## Abstract

The extracellular matrix (ECM) and membrane proteolysis play a key role in structural and functional synaptic plasticity associated with development and learning. A growing body of evidence underscores the multifaceted role of members of the metzincin superfamily, including metalloproteinases (MMPs), A Disintegrin and Metalloproteinases (ADAMs), A Disintegrin and Metalloproteinase with Thrombospondin Motifs (ADAMTSs) and astacins in physiological and pathological processes in the central nervous system (CNS). The expression and activity of metzincins are strictly controlled at different levels (e.g., through the regulation of translation, limited activation in the extracellular space, the binding of endogenous inhibitors and interactions with other proteins). Thus, unsurprising is that the dysregulation of proteolytic activity, especially the greater expression and activation of metzincins, is associated with neurodegenerative disorders that are considered synaptopathies, especially Alzheimer’s disease (AD). We review current knowledge of the functions of metzincins in the development of AD, mainly the proteolytic processing of amyloid precursor protein, the degradation of amyloid β (Aβ) peptide and several pathways for Aβ clearance across brain barriers (i.e., blood-brain barrier (BBB) and blood-cerebrospinal fluid barrier (BCSFB)) that contain specific receptors that mediate the uptake of Aβ peptide. Controlling the proteolytic activity of metzincins in Aβ-induced pathological changes in AD patients’ brains may be a promising therapeutic strategy.

## Introduction

Specific proteolysis of the extracellular matrix (ECM) and membrane proteins in close proximity to the synapse emerged as an important mechanism of synaptic plasticity, learning and memory. Extracellular proteases are ubiquitously expressed in neurons and glial cells throughout the brain. Among more than 500 proteases that are encoded in the mammalian genomes (Puente et al., 2003), numerous members of the metzincin superfamily, including metalloproteinases (MMPs), A Disintegrin And Metalloproteinases (ADAMs), A Disintegrin And MMPs with Thrombospondin Motifs (ADAMTSs), and astacins, are implicated in the regulation of various aspects of plasticity in diverse neurons and synapses (Bajor and Kaczmarek, 2013). For example, MMP-9 plays a key role in the structural plasticity of dendritic spines (Michaluk et al., 2011; Dziembowska and Wlodarczyk, 2012). MMP-3 controls long-term potentiation (LTP) that depends on L-type calcium channels (Wiera et al., 2017). Additionally, ADAM-10 and ADAM-17 regulate the induction of long-term depression (LTD; Cho et al., 2008; Marcello et al., 2013). Consequently, together with a number of their perisynaptic substrates, secreted and membrane proteases influence synapses by interfering with cell-cell connections (e.g., trans-synaptic or neuron-astrocyte contacts) and cell-ECM adhesion.

In addition to the high number of encoded proteases and several different types of these enzymes, yet another feature of proteases is their multifunctionality that depends on the context in which they are involved in biological activity. Indeed, the same metzincins that play a key role in developmental processes or other physiological synaptic mechanisms have been implicated in synaptic degeneration under pathological conditions. Uncontrolled excess or deficiency of the extracellular or membrane proteolysis is often implicated in the etiology of many neurodegenerative disorders that are considered synaptopathies, such as Alzheimer’s disease (AD) and epilepsy (Palop and Mucke, 2010; Bronisz and Kurkowska-Jastrzebska, 2016). Thus, a detailed understanding of the mechanisms that regulate the expression and activity of different membrane and extracellular proteases and their substrates at synapses is needed to fully understand the pathological mechanisms that underlie synaptic dysfunctions that are related to such neurodegenerative disorders.

In the present review article, we focus on the mechanisms that underlie the involvement of synaptic proteolysis in physiological brain functions and neurodegeneration, especially in AD. We first provide a brief overview of the main functions of extracellular proteolysis in neuronal tissue. We then summarize the current understanding of the roles that are played by different membrane and extracellular metzincins that are present at synapses or in the perisynaptic area that control synaptic plasticity and learning. Next, we focus on the mechanisms that regulate the proteolytic processing of amyloid precursor protein (APP) and the catabolism of amyloid β (Aβ) peptide in AD. Finally, we discuss the roles of extracellular proteases as fine regulators of Aβ clearance and the permeability of brain barriers. We emphasize that interactions between metzincins and the blood-cerebrospinal fluid barrier (BCSFB) and blood-brain barrier (BBB), which may open promising avenues into new therapeutic interventions for the treatment of AD.

### General Functions of Extracellular Proteolysis in the Brain

Extracellular proteolysis in the brain can broadly be described in terms of five processes, related to the cleavage, processing, turnover, remodeling or degradation of the target protein. These processes may partially overlap (Apte and Parks, 2015).

(1) *Cleavage* generally refers to limited proteolysis at specific sites (usually a single site) of the target protein. The proteases cleave membrane proteins, often resulting in the release of soluble truncated forms with signaling properties. Proteases that belong to the ADAM and MMP families are responsible for the controlled cleavage of membrane proteins, referred to as shedding. Among the ADAMs, two neuronal proteases (ADAM-10 and ADAM-17, also known as tumor necrosis factor-α-converting enzyme (TACE)) mediate the shedding of ectodomains from membrane adhesion proteins or receptors. This process is followed by secondary cleavage that is mediated by γ-secretase (see “Role of Metzincins in APP Cleavage” Section). For example, ADAM-10 cleaves neuronal APP and releases a soluble domain called sAPPα that affects the induction of LTP and learning (Ring et al., 2007). Additionally, on the cytoplasmic side, the intracellular domains that are produced by the activity of γ-secretase often show nuclear activity (e.g., intracellular fragment of N-cadherin, adhesion protein L1, or telencephalin; Bajor and Kaczmarek, 2013). In addition to membrane proteins, also the ECM is a source of bioactive fragments called matricryptins or matrikines that are produced upon proteolytic cleavage (Ricard-Blum and Vallet, 2016). Some matricryptins that are released from the ECM are present in the brain where they regulate synaptic functions (Wang T. et al., 2014). Other matricryptins may be involved in brain pathophysiology. For example, the ectodomain of collagen XVIII (called endostatin) or matricryptin that is released from collagen XXV binds to Aβ peptide and inhibits the formation of amyloid fibrils *in vitro* (Osada et al., 2005). Altogether, the majority of proteases that decrypt the brain ECM belong to the metzincin superfamily (Ricard-Blum and Vallet, 2016).

(2) *Processing* mainly leads to the proteolytic activation of a latent protein. Pro-brain-derived neurotrophic factor (BDNF) that is secreted from neurons binds to the p75 receptor and promotes cell death. However, after the proteolytic processing of pro-BDNF that removes the propeptide sequence, mature BDNF binds and activates the tropomyosin receptor kinase B (TrkB) receptor, affecting LTP induction and learning (Edelmann et al., 2015). The tissue plasminogen activator (tPA)-plasmin system or MMP-9 is responsible for the proteolytic processing of BDNF (Nagappan et al., 2009; Mizoguchi et al., 2011a). Extracellular proteolysis may also activate pro-forms of proteases. In particular, the activation of the majority of extracellular proteases requires proteolytic processing of the inhibitory pro-domain. tPA is responsible for the conversion of plasminogen to active plasmin (Yepes et al., 2009). Another protease that is abundantly expressed in the brain, MMP-9, is activated by other metzincins or cathepsin B that is released from lysosomes to the extracellular space (Van den Steen et al., 2001; Padamsey et al., 2017). Proteolytic processing may also activate membrane receptors. Protease-activated receptor-1 (PAR-1) belongs to the G protein-coupled receptor family and is activated through extracellular proteolysis. PAR-1 activation occurs during *N*-methyl-D-aspartate (NMDA) receptor-dependent memory formation and synaptic plasticity (Almonte et al., 2013).

(3) *Turnover* is responsible for the breakdown and replacement of target proteins and is thus considered a purely homeostatic process. Proteoglycans are the most abundant group of ECM proteins in the brain. They form characteristic structures called perineuronal nets. These proteins have long half-lives, up to years, and thus slow turnover (Tsien, 2013). In contrast, membrane proteins may be constitutively cleaved and replaced. For example, APP undergoes constitutive or regulated α-secretase cleavage that is mediated by ADAM-10, among others (Lammich et al., 1999; see “Role of Metzincins in APP Cleavage” Section). Generally, proteases that are responsible for protein turnover are involved in controlling the spatial distribution and level of extracellular and membrane proteins.

(4) *Remodeling* is associated with changes in ECM structure or ECM-cell interactions and is related to normal physiological proteolysis, followed by the clearance of target protein. Certain synaptic adhesive complexes have an extremely high affinity association constant that prevents their activity-dependent dissociation under physiological conditions. In this case, the proteolysis of such complexes may regulate the lifetime of these structures and give rise to the appearance of cleavage products with various signaling properties. Trans-synaptic interactions between the Eph receptor and ephrins and the proteolytic remodeling of this complex by neuropsin or MMPs are one example (Himanen et al., 2007). Proteases may also modulate the kinetics, specificity and duration of signals that are produced by receptor-ligand interactions. For example, MMPs and ADAMs control axonal growth and pathfinding by cleaving axon guidance receptors and ligands (repulsive or attractive; Bai and Pfaff, 2011). Notably, despite several newly discovered roles for extracellular proteolysis, its most intuitive function remains direct reorganization of the ECM structure. The local proteolysis of laminin, fibronectin and proteoglycans in a small volume of neuropil in the vicinity of synapses may locally loosen the ECM structure, thus allowing plasticity-related structural changes to, for example, dendritic spines. Accordingly, Tsien (2013) proposed an interesting hypothesis, in which engrams may be partially encoded in the network of local “holes” in perineuronal nets and the ECM, which can be produced by proteolysis.

(5) *The inactivation or degradation* of proteins and peptides through proteolysis leads to the removal of a particular protein or its biological activity. The unregulated or excessive degradation of proteins in the extracellular space is often observed under pathological conditions, such as neuroinflammation and neurodegeneration (Rosenberg, 2009). Inactivation through proteolysis may have beneficial consequences. For example, Aβ peptide in the form of monomers or oligomers is degraded by different proteases (see “Proteolytic Degradation of Aβ by Metzincins” Section). For instance, MMP-9 is able to cleave and inactivate Aβ fibrils and amyloid plaques (Yan et al., 2006).

### Metzincin Clan of Proteases

The human and mouse genomes encode more than 500 proteases, representing >1.5% of protein-coding genes (Puente et al., 2003). All proteases are divided into six superfamilies, based on the nucleophile that participates in the mechanism of peptide bond cleavage: serine, threonine, cysteine, aspartic acid, metallo and glutamic acid. MMPs (or metalloendopeptidases; EC subclass 3.4.24) contain catalytically essential zinc cation that is coordinated by histidines and acidic residue. Among the clan of metalloendopeptidases, one may distinguish the superfamily of zincins that contains the HEXXH sequence embedded in an active site that is responsible for positioning the catalytic zinc. Glutamate residue that is present in this sequence binds and polarizes water molecule that play a role in peptide bond cleavage. Zincins are further subdivided into clans with metzincins. Metzincins contain two characteristic features: (1) an HEXXHXXXGXXH/D sequence that coordinates catalytic zinc; and (2) a highly conserved 1,4-β-turn that contains a methionine residue and thus is called a Met-turn, thus indicating the name of the clan (Gomis-Rüth, 2003). Metzincins in the mammalian genome comprise a few different families, including astacins, leishmanolyisn (with single leishmanolysin-2), MMPs (matrixins), ADAMs and ADAMTSs.

In contrast to proteases that are responsible for protein digestion and degradation, mammalian metzincins are mainly involved in proteolytic processing. Metzincins usually cleave a limited number of peptide bonds at specific and restricted sites to activate, modulate, or inactivate target protein functions. Thus, metzincins may be regarded as regulatory proteases with signaling properties. Metzincins are either secreted or associated with the membrane (through a transmembrane segment or glycosylphosphatidylinositol [GPI] anchor), and they are expressed as pre-pro-proteins. The pre-sequence guides the protease to the secretory pathway, and the pro-peptide blocks the activity of zymogen. With a few exceptions in the ADAMTS family, pro-peptide is removed from the catalytic site to activate the protease by convertases (e.g., furin) or autocatalytically during transit through the Golgi apparatus (Weber and Saftig, 2012).

**Figure 1:**
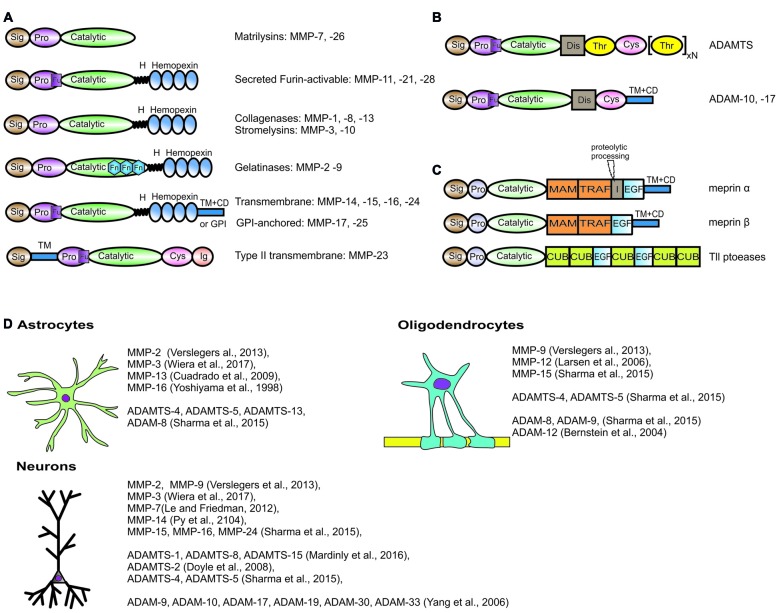
Domain structure and brain expression of different metzincins described in the review article. The domain organization of metalloproteinases (MMPs) **(A)**, adamlysins **(B)** and astacins **(C)**. Sig, signal peptide; Pro, propeptide; Fu, Pro-protein convertase (furin) cleavge site; H, hinge region; Fn, fibronectin type II motif; TM + CD, transmembrane type I domain with cytoplasmic domain; GPI, Glycosylphosphatidylinositol-anchoring sequence; Cys, cysteine-rich domain; Ig, immunoglobulin-like domain; Dis, disintegrin domain; Thr, thrombospondin type I-like domain; MAM, meprin/A5-protein/PTPmu domain; TRAF, MATH/TRAF domain; I, inserted domain; EGF, epidermal growth factor-like domain; CUB, complement C1r/C1 s, Uegf, Bmp1 domain. Meprin α contains proteolytic processing sequence in inserted domain, that is essential for the production of soluble meprin α if not coexpressed with meprin β. **(D)** List of metzincins that are confirmed to be expressed by oligodendrocytes, astrocytes and neurons in the central nervous system (CNS).

Typical members of the MMP family comprise (from the N-terminus) a signal peptide, a pro-sequence with a cysteine switch motif that maintains the enzyme in an inactive state (proMMP), a zinc- and calcium-dependent catalytic domain, a linker region, and a hemopexin C-terminal domain (Figure 1A). They are either secreted from the cell or anchored to the plasma membrane. Based on sequence similarity, substrate specificity and domain organization, MMPs are usually classified into collagenases that cleave the triple helix of a native collagen (MMP-1, MMP-8, MMP-13), gelatinases that target gelatin—the denatured collagen (MMP-2, MMP-9), stromelysins that cleave ECM proteoglycans (MMP-3, MMP-10, MMP-11), matrilysins (MMP-7, MMP-26), membrane-type MMPs with a transmembrane type I domain (MMP-14, MMP-15, MMP-16, MMP-24) and membrane-type MMPs with a GPI anchor (MMP-17, MMP-25), among others (Visse and Nagase, 2003).

The members of the ADAM family comprise (from the C-terminus) a cytoplasmic domain, a transmembrane domain, an epidermal growth factor-like domain and/or cysteine-rich domain, a disintegrin domain and a protease domain. Nevertheless, not all ADAMs possess proteolytic activity (Figure 1B). Among active ADAMs (called sheddases), ADAM-8, ADAM-9, ADAM-10, ADAM-12, ADAM-15, ADAM-17, ADAM-19, ADAM-20, ADAM-21, ADAM-28, ADAM-30 and ADAM-33 are expressed in humans.

All ADAMTSs are secreted and operate in the extracellular space. The domain organization of ADAMTS comprises (from the N-terminus) a signal peptide, a pro-domain, a catalytic protease domain, a disintegrin-like domain, a thrombospondin repeat motif and a cysteine-rich domain followed by a spacer region (Kelwick et al., 2015). The majority of ADAMTSs also contain C-terminal ancillary domains that differ between family members and regulate associations with ECM structures or the binding of particular substrates. Mammalian ADAMTS may be further subdivided into subclasses based on domain composition and cleavage specificity. ADAMTS-1, ADAMTS-4, ADAMTS-5, ADAMTS-8, ADAMTS-9, ADAMTS-15 and ADAMTS-20 can efficiently cleave chondroitin sulfate proteoglycans; therefore, they are called aggrecanases or proteoglycanases. In contrast, ADAMTS-2, ADAMTS-3 and ADAMTS-14 are responsible for processing triple helical collagens; therefore, they belong to the pro-collagen N-propeptidase group.

The human and mouse genomes have six genes that encode members of the astacin family, including two meprins (α and β), bone morphogenetic protein-1 (BMP1) and two tolloid-like (Tll) proteases and ovastacin. The domain organization and overall structure of astacins have been the subject of an extensive review (Figure 1C; Gomis-Rüth et al., 2012).

Importantly, zymogen that is synthesized within the cell and released into the extracellular space usually undergoes several steps to accomplish its functional role, including activation, targeting the enzyme to the proper extracellular or membrane compartment that provides access to the substrate, modulation by cofactor binding, inactivation by endogenous protein inhibitors and the route of internalization. All of these processes comprise a complex repertoire of mechanisms that control the activity of metzincins. Failures of these regulatory mechanisms can give rise to several pathologies, such as neuroinflammation, AD and multiple sclerosis. Indeed, several brain disorders are characterized by the excessive expression or activity of proteolytic enzymes (Rosenberg, 2009). On the other hand, nowadays, an extensive body of evidence clearly demonstrates that the activity of different metzincins underlies many physiological processes in the brain, including the formation of memory traces and cognition.

### Metzincins in the Brain: Expression and Roles in Synaptic Plasticity and Learning

In the following sections, we provide an overview of our current knowledge of the expression of different metzincins in the brain and their roles in synaptic plasticity and learning (Figure 1D). We first discuss different metzincins that are known to be engaged in the regulation and maintenance of synaptic plasticity and related cognitive processes. We then outline the current knowledge of the roles of these enzymes in the progression and treatment of AD and related pathologies.

Among metzincins, MMP-9 is by far the most widely studied protease in the context of synaptic plasticity, learning, and memory (Dziembowska et al., 2013; Kaczmarek, 2016). Gene knockout or inhibition of MMP-9 in mice blocks the maintenance of LTP at different synapses in the hippocampus (Nagy et al., 2006; Wiera et al., 2013; Wójtowicz and Mozrzymas, 2014), amygdala (Gorkiewicz et al., 2015), and cerebral cortex (Okulski et al., 2007; Lebida and Mozrzymas, 2016). MMP-9-deficient mice also exhibit impairments in plasticity in the somatosensory (Kaliszewska et al., 2012) and visual (Spolidoro et al., 2012) cortex after whisker or light deprivation respectively. Additionally, MMP-9 knockout mice exhibit impairments in contextual fear conditioning (Nagy et al., 2006), inhibitory avoidance learning (Nagy et al., 2007) and appetitive amygdala-dependent learning (Knapska et al., 2013).

The induction of LTP and learning (e.g., contextual fear conditioning) lead to an increase in proMMP-9 production and MMP-9 proteolytic activity in the hippocampus, cortex, and amygdala (Wiera et al., 2012; Ganguly et al., 2013; Lebida and Mozrzymas, 2016). Latent proMMP-9 is locally translated in dendrites (Dziembowska et al., 2012) in a process that depends on fragile X mental retardation 1 protein (Janusz et al., 2013; Sidhu et al., 2014), eukaryotic initiation factor 4E (Gkogkas et al., 2014) and miR-132 (Jasińska et al., 2016). Higher MMP-9 activity appears as soon as 10 min after an episode of increased neuronal activity *in vitro* (Stawarski et al., 2014a). After a short period of activity, MMP-9 is inhibited by endogenous tissue inhibitor of MMPs-1 (TIMP-1; Magnowska et al., 2016) and internalized by lipoprotein receptor-related protein 1 (LRP1; Etique et al., 2013).

The role of MMP-9 in the structural plasticity of dendritic spines has been extensively studied (Stawarski et al., 2014b). The effect of MMP-9 on the morphology of dendritic spines depends on the duration of MMP exposure. Short-term application of MMP-9 or its application followed by inhibition induces the maturation of dendritic spines (Wang et al., 2008; Magnowska et al., 2016). Whereas, the extended application of exogenous recombinant active MMP-9 leads to the elongation of dendritic spines (Bilousova et al., 2009; Michaluk et al., 2011). Moreover, the prolonged neuronal overexpression of MMP-9 in rats negatively affects LTP induction in the hippocampus (Wiera et al., 2013, 2015; Magnowska et al., 2016) and increases the severity of kainate-induced epilepsy (Wilczynski et al., 2008).

**Figure 2:**
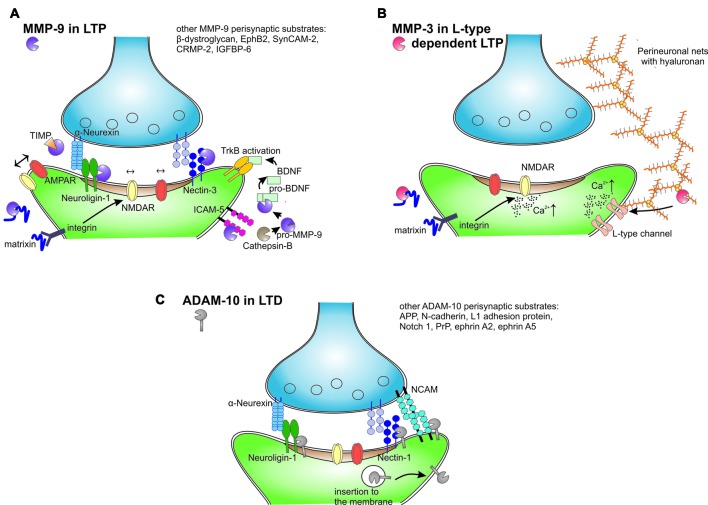
Extracellular and membrane proteolysis mediated by different metzincins in the synaptic plasticity. **(A)** MMP-9 released from neurons after an episode of increased neural activity is activated in extracellular space by cathepsin B. MMP-9 cleaves perisynaptic neuroligin-1, nectin-3, intercellular adhesion molecule 5 (ICAM-5) and unrecognized matrix proteins to release matrixins that activate integrin receptors and modulate lateral movement and synaptic trapping of NMDA and α-amino-3-hydroxy-5-methyl-4-isoxazolepropionic acid (AMPA) receptors. MMP-9 activates also pro-brain-derived neurotrophic factor (BDNF) to mature BDNF that activates tropomyosin receptor kinase B (TrkB) receptor in autocrine fashion. After short period of activity MMP-9 is inhibited by TIMPs and internalized. **(B)** MMP-3 is selectively involved in LTP that is dependent on the activation of L-type voltage-gated calcium channels. MMP-3 is able to cleave almost all brain proteoglycans and other protein constituents of perineuronal nets. Hypothetically, MMP-3 activity releases peptides or domains that in turn affect the L-type channels and modulate the induction of LTP. MMP-3 affects also integrin signaling and calcium influx through NMDA receptors. **(C)** ADAM-10 in synaptic plasticity. LTP decreases ADAM-10 synaptic levels through increased endocytosis. Conversely, induction of long-term depression (LTD) leads to the insertion of ADAM-10 to the membrane in a mechanism that is dependent on synapse-associated protein 97 (SAP97). LTD stimulates ADAM-10 cleavage of amyloid precursor protein (APP), neuroligins and other adhesion proteins.

Numerous putative MMP-9 substrates were revealed by proteomic mass spectrometry studies *in vitro*. However, a limited number of brain MMP-9 substrates have been described in the context of learning (Figure 2A). The best documented neuronal substrates of MMP-9 are neuroligin-1 (Peixoto et al., 2012), nectin-3 (van der Kooij et al., 2014), pro-BDNF (Mizoguchi et al., 2011a), β-dystroglycan (Michaluk et al., 2007; Ganguly et al., 2013; Knapska et al., 2013) and intercellular adhesion molecule 5 (ICAM-5; Conant et al., 2011; Kelly et al., 2014).

The synaptic function of MMP-9 is well documented for the cleavage of neuroligin-1. Light deprivation during the critical period enhances synaptic plasticity in the visual cortex, and this process is accompanied by an increase in the proteolysis of neuroligin-1 that depends on MMP-9, NMDA receptors and Ca^2+^/calmodulin-dependent protein kinase (CaMKII; Peixoto et al., 2012). As a result, neuroligin-1 cleavage transiently destabilizes the trans-synaptic nauroligin-neurexin complex during plasticity-related synaptic remodeling.

The effect of perisynaptic MMP-9 activity strongly depends on integrin signaling. The inhibition of integrin β1 abolishes the effect of MMP-9 on NMDA receptor lateral membrane diffusion (Michaluk et al., 2009), prevents the increase in the size of dendritic spines after MMP-9 application (Wang et al., 2008), and blocks the increase in synaptic strength after MMP-9 administration (Nagy et al., 2006). It has been proposed that MMP-9 may associate with α5 and β integrins (Piccard et al., 2007) to focus enzymatic activity where it is required. The interplay between MMP-9 and integrins may also be explained by the MMP-9-dependent cleavage of certain membrane or ECM proteins to release bioactive matricryptins (Ricard-Blum and Vallet, 2016). These peptides bind and activate integrin receptors, leading to cytoskeletal reorganization in dendritic spines, the exocytosis of α-amino-3-hydroxy-5-methyl-4-isoxazolepropionic acid (AMPA) receptors, remodeling of the postsynaptic density, and the functional strengthening of synapses (for review, see Huntley, 2012). Conversely, different MMPs cleave specific ECM proteins to release distinct matricryptins. Endostatin is a proteolytically released fragment of collagen XV/XVIII that signals trans-synaptically during homeostatic plasticity in *Drosophila* neuromuscular junctions (Wang T. et al., 2014). Another released matricryptin of collagen XIX promotes the assembly of inhibitory nerve terminals through integrin receptors (Su et al., 2016). All of the aforementioned collagens are cleaved not only by MMP-9 but also by MMP-3, MMP-7, MMP-13 and MMP-20, among others (Ricard-Blum and Vallet, 2016). Thus, future studies should unravel the whole complex proteome of matricriptins in relation to the activity of specific metzincins in synapses undergoing plastic changes.

Presynaptic β1 integrin binds ICAM-5 adhesion protein on immature filopodia tips during early stages of synaptic formation and inhibits the filopodia-to-spine transition (Ning et al., 2013). The extracellular domain of ICAM-5 is cleaved by several metzincins (e.g., MMP-9 and ADAMs) during spine maturation, the induction of LTP and learning (Tian et al., 2007; Conant et al., 2010, 2011; Niedringhaus et al., 2012; Kelly et al., 2014). Another cell adhesion molecule, nectin-3, is processed by MMP-9 during chronic stress-induced plasticity in hippocampus (van der Kooij et al., 2014). Thus, one of the main effects of MMP-9 activity in the neuropil is the cleavage of transsynaptic and perisynaptic cell adhesion proteins that leads to structural and functional synaptic reorganization during synaptic plasticity and learning.

As indicated in Supplementary Table S1, MMPs beyond MMP-9 are also expressed in the mouse and human brains. However, our knowledge of the physiological roles of these MMPs in the brain is limited. The lack of specificity of available MMP blockers delimits unequivocal interpretation of experiments. For example, nonspecific MMP inhibitors (e.g., GM-6001 and NNGH, which block both MMP-9 and other MMPs, ADAMs and ADAMTSs) impair early- and translation-dependent late-LTP phases (Meighan et al., 2007; Conant et al., 2010), excitatory postsynaptic potential-to-spike potentiation (Wójtowicz and Mozrzymas, 2014), the plasticity of NMDAergic transmission (Brzdak et al., 2017), the structural plasticity of dendritic spines (Szepesi et al., 2014), ocular dominance plasticity (Spolidoro et al., 2012) and memory formation (Meighan et al., 2006). Moreover, almost all of the commercially available inhibitors of metzincins block at least some MMPs and/or ADAMs. Thus, attempts to attribute specific roles to the activity of different metzincins in distinct plasticity phenomena remain a major challenge in this field, especially when experiments are based on pharmacological tools. Studies that use knockout animals should help resolve this issue, but careful tests of possible compensatory processes are necessary. There are numerous examples of compensatory changes that occur in MMP-deficient mice. For example, in MMP-2 and MT1-MMP knockout mice, compensatory MMP-9 upregulation occurs in lymphocytes that accounts for the observed phenotypes (Esparza et al., 2004; Hsu et al., 2006). Additionally, MMP-3 knockout mice exhibit a compensatory increase in MMP-7 and MMP-12 expression during colonic mucosal hyperplasia (Li et al., 2004). Similarly, MMP-9 deletion leads to a compensatory increase in MMP-8 expression, at least in the myocardium (Chiao et al., 2012). Thus, the physiological relevance of other MMPs in the brain should be confirmed using specific loss-of-function studies with deficient or knockdown animals, accompanied by a thorough examination of possible compensatory mechanisms. Additionally, the structural similarity between MMPs reduces the specificity and sensitivity of antibodies against different proteases. Some MMP antibodies, for example, may recognize TIMPs or fibronectin (De Groef et al., 2015), thus making studies that are based on a single antibody highly uncertain.

#### MMP-3

The functions of MMP-3 in different neuropathologies, during brain development, or in adult CNS have only been partially unraveled (for review see Van Hove et al., 2012a). The expression of MMP-3 mRNA is upregulated in the hippocampus after tetanus-induced epilepsy (Gorter et al., 2007) and trauma-induced synaptogenesis (Kim et al., 2005). Similarly, inhibitory avoidance conditioning and spatial learning in the water maze increase the levels of zymogen and active MMP-3 (Meighan et al., 2006; Olson et al., 2008). In addition, deficits in the formation of associative memory were observed after administration of an MMP-3 inhibitor (Wright et al., 2006). The role of MMP-3 in neuroplasticity is further strengthened by studies in MMP-3-deficient mice, in which impairments in motoneuronal endplate remodeling were observed after denervation (Chao et al., 2013), in addition to impairments in motor coordination, motor learning and balance (Van Hove et al., 2012b). Moreover, MMP-3-null mice exhibit substantial impairments in cross-modal plasticity in the visual cortex after monocular enucleation (Aerts et al., 2015) and deficits in the maintenance of LTP in the hippocampus (Wiera et al., 2017). In the hippocampal CA3-CA1 projection, two distinct components of LTP that depend on NMDA receptors or L-type voltage-gated calcium channels are present. Interestingly, the activation of MMP-3 appears to specifically support L-type channel-dependent LTP (Figure 2B), whereas NMDA-dependent LTP is solely contingent upon MMP-9 activity (Wiera et al., 2017). Future studies that use models of MMP-3 deficiency should further unravel the mechanisms by which MMP-3 regulates synaptic plasticity and learning. Another unresolved issue is the way in which MMP-3 and MMP-9 proteases are activated and retained at the perisynaptic space during LTP and learning. Nitric oxide may play an important role in this process (Manabe et al., 2005).

#### MT-MMPs

The abundant expression of membrane MMPs in the CNS (Supplementary Table S1) prompted investigations on their potential roles in neurotransmission and synaptic plasticity. MT1-MMP (MMP-14) is required for the activation of proMMP-2 and proMMP-9 (Toth et al., 2003) and thus may regulate the time window of MMP-9 perisynaptic activity. The role of MT1-MMP in synaptic plasticity is further strengthened by the upregulation of this protease in one-trial odor learning in the mouse amygdala and hippocampus (Irwin and Byers, 2012). Other MT-MMPs (e.g., MMP-16 and MMP-17) are expressed in the basolateral nucleus of the amygdala where they colocalize with CaMKII in neurons. The conversion of pro-BDNF to the mature form by both proteases was postulated during LTP induction in the amygdala (Li C. et al., 2011). Likewise, the involvement in synaptic functions was also clearly demonstrated for MT5-MMP (MMP-25). This protease is mainly expressed in the adult brain, and its high levels were reported in the cerebellum and hippocampal formation (Jaworski, 2000). MT5-MMP is also enriched in synapses through interactions with proteins that contain PDZ domains, such as glutamate receptor interacting protein (GRIP) and AMPA receptor binding protein (ABP; Monea et al., 2006). Willem et al. (2015) reported that MT5-MMP cleaved APP protein in a sequence that was located upstream of the β-secretase cleavage site. After this event, the α-secretase ADAM-10 generated a peptide, called Aη-α, that inhibited the induction of LTP. Therefore, one possibility is that MT5-MMP, in concert with other proteases, could be responsible for the shedding of synaptic membrane proteins also in physiological brain processes.

#### Other MMPs

The brain functions of other soluble MMPs have not been studied extensively, but some evidence suggests the involvement of MMP-1, MMP-7, MMP-12 and MMP-13 in plasticity-related phenomena. The upregulation of active MMP-13 was observed in neuronal nuclei and astrocytes after ischemia-induced plasticity (Cuadrado et al., 2009; Lu et al., 2009). Similarly, the expression of MMP-12 increased after bicuculline-induced chemical LTP (Pegoraro et al., 2010). Additionally, MMP-12-deficient mice exhibited a delay in myelination of the optic nerve because of a lack of insulin-like growth factor (IGF) binding protein 6 cleavage that regulates IGF-2 bioavailability (Larsen et al., 2006), a protein that is highly important for LTP induction and learning (Chen et al., 2011; Stern et al., 2014). MMP-1 is also expressed at low levels in the brain but is upregulated after an increase in neuronal activity that is induced by kainate treatment (Ierusalimsky and Balaban, 2013) or after ischemia (Lenglet et al., 2014). Endogenous MMP-1 induces neuronal Ca^2+^ influx through PAR-1 activation (Boire et al., 2005). Moreover, transgenic mice that overexpress MMP-1 in astrocytes exhibit alterations in anxiety and deficits in sociability and spatial learning (Allen et al., 2016). A few studies indicate a synaptic role for MMP-7. For example, the application of exogenous MMP-7 in neuronal cultures impaired synaptic vesicle release (Szklarczyk et al., 2007), resulting in the elongation of dendritic spines (Bilousova et al., 2006) and ectodomain cleavage of the NMDA receptor (Szklarczyk et al., 2008). Further studies are needed to elucidate the relationship between the expression of these MMPs in the CNS and their precise roles in synaptic physiology.

#### ADAMTS

MMPs have been considered the main family of matrix-degrading proteases. Nonetheless, detailed studies have shown that MMPs work more as limited-cleavage proteases that mainly control cell signaling. In contrast, other metzincins are responsible for ECM maturation and constitutive turnover. Numerous ADAMTS proteinases are present in the CNS, including the cortex, hippocampus, and spinal cord (Supplementary Table S2) where they are involved in tissue development, turnover and maintenance (Kelwick et al., 2015). However, strong evidence also indicates the involvement of ADAMTSs in neuronal plasticity. At least two proteoglycanases, ADAMTS-1 and ADAMTS-4, are able to cleave the chondroitin sulfate proteoglycan brevican, releasing a characteristic truncated fragment that was found in the hippocampus (Nakamura et al., 2000; Yuan et al., 2002). Additionally, lower levels of synaptic proteins were reported in female ADAMTS-1-null mice, suggesting a role for ADAMTS-1 in developmental plasticity and synaptogenesis (Howell et al., 2012). Interestingly, the activity-dependent expression of ADAMTS-1 mRNA was found exclusively in somatostatin positive interneurons (Mardinly et al., 2016). However, in adult ADAMTS-1-null mice, learning and spatial memory were unaffected in the radial-arm water maze (Howell et al., 2012).

Learning and memory have not been tested in mice that are deficient in other ADAMTSs that are expressed in the brain. ADAMTS-4, ADAMTS-5 and ADAMTS-15 deserve special attention. A recent study described the expression of the proteoglycanases ADAMTS-4 and ADAMTS-15 in the brain (Levy et al., 2015). Both of these proteases are expressed in the hippocampus and neocortex, but they are produced by distinct cells. ADAMTS-15 is generated by parvalbumin-expressing, fast-spiking interneurons that are ensheathed by perineuronal nets, whereas ADAMTS-4 is produced exclusively by oligodendrocytes, with peak expression at early stages of postnatal development during the critical period (Levy et al., 2015). Brain proteoglycans and their digestion by exogenous enzymes markedly influence synaptic plasticity and learning (Sorg et al., 2016). An interesting line of investigation would be to determine whether some ADAMTSs may be responsible for the turnover and activity-dependent remodeling of perineuronal nets and brain proteoglycans.

#### ADAMs

Among enzymatically active ADAMs, ADAM-8, ADAM-9, ADAM-10, ADAM-12, ADAM-15, ADAM-17, ADAM-19 and ADAM-33 are expressed in the CNS during development or in the adult brain (Supplementary Table S3). Evidence suggests that two representatives of this family, ADAM-10 and ADAM-17, play a role in synaptic plasticity. ADAM-10 expression in the brain is rather widespread, where it operates as a constitutively active α-secretase that is responsible for the physiological turnover of membrane proteins (Saftig and Lichtenthaler, 2015). It cleaves APP within the Aβ sequence, thus preventing the formation of amyloidogenic peptides. In addition to APP, ADAM-10 cleaves numerous synaptic adhesion proteins, including neural cell adhesion molecule (NCAM; Brennaman et al., 2014), nectin-1 (Kim et al., 2010); neuroligin-1 (Suzuki et al., 2012) and N-cadherin (Malinverno et al., 2010), among others.

ADAM-10 is present in the postsynaptic density of excitatory synapses (Malinverno et al., 2010) through interactions with the scaffold protein synapse-associated protein 97 (SAP97; Marcello et al., 2007). The level of synaptic ADAM-10 is controlled in an activity dependent manner. The induction of LTP in neuronal cultures promotes ADAM-10 endocytosis and thus decreases its synaptic activity. Conversely, LTD induces ADAM-10 membrane insertion and stimulates its activity in hippocampal slices (Figure 2C; Marcello et al., 2013). As a result, SAP97-dependent ADAM-10 trafficking to synapses is necessary for LTD induction and the structural plasticity of dendritic spines (Marcello et al., 2013). Classic ADAM-10 knockout in mice is prenatally lethal; therefore, conditional deletion was necessary to assess protease function in the adult brain. The loss of ADAM-10 in mature neurons impaired spatial learning, caused the complete loss of LTP in the CA3-CA1 hippocampal pathway, and increased seizure susceptibility (Prox et al., 2013). In contrast, ADAM10 overexpression in transgenic mice enhanced synaptogenesis (Bell et al., 2008) and alleviated LTP and learning deficits in an AD model (Postina et al., 2004).

Another member of the ADAM family, ADAM17, is required for metabotropic glutamate receptor-dependent LTD (Cho et al., 2008). The induction of this form of plasticity is associated with the ADAM-17-mediated cleavage of neuronal pentraxin (NPTX), which binds to and clusters AMPA receptors. After NPR cleavage, AMPA receptors are endocytosed, and synaptic LTD is expressed (Cho et al., 2008). Additionally, unrecognized metzincin protease is responsible for the cleavage of postsynaptic adhesion molecule netrin-G ligand-3 during the induction of LTD in the hippocampus (Lee et al., 2013). The inhibition of all MMPs examined to date has been shown to not affect LTD induction (Nagy et al., 2006). Therefore, ADAM10 or ADAM17 likely cleaves synaptic netrin-G ligand-3.

#### Astacins

Among six members of the astacin family that are expressed in the mammalian genome, at least three are expressed in the CNS (Supplementary Table S3): ovastacin, meprin α and BMP1. The functions of astacins in the brain have not been extensively studied. Meprin α and Tll proteases deserve more in-depth investigation. Meprin α processes proMMP-9 and enhances its activation kinetics (Geurts et al., 2012). Additionally, meprin α is able to cleave proteins that are related to synaptic plasticity, such as Reelin (Sato et al., 2016) and vasoactive intestinal peptide (Sterchi et al., 2008). In addition, studies in non-mammalian species have demonstrated a role for Tll proteases in learning. Indeed, Tll-1 mRNA is upregulated during classical eye blink conditioning in turtles (Keifer et al., 2009). Furthermore, a tolloid-like gene in Aplysia is regulated during non-associative long-term sensitization (Liu et al., 1997). However, the precise role of Tll-1 and Tll-2 (and other astacins) in the mammalian brain remains unknown.

## Expression of Metzincins in Alzheimer’S Disease

In the second part of this review article, we provide a concise description of our current knowledge of the role of metzincins in the pathogenesis of the most common dementing disorder, AD. At the cellular level, the majority of pathological manifestations of AD are related to synapses; thus, AD may be considered a synaptopathy (Palop and Mucke, 2010). Pathological elevations of Aβ peptide levels lead to the partial blockade of NMDA receptors, resulting in insufficient NMDA receptor activation for LTP induction (Shankar et al., 2007). This effect results in a shift in the activation of NMDA receptor-dependent signaling toward the preferential induction of LTD rather than LTP and the loss of synapses. Compromised LTP and augmented LTD are commonly observed in mouse models of AD (Li et al., 2009; Marchetti and Marie, 2011). Although the precise mechanisms by which Aβ peptide induces LTD are not fully understood, but it is known that it depends on AMPA and NMDA receptor internalization and the disappearance of dendritic spines (Snyder et al., 2005; D’Amelio et al., 2011; Reinders et al., 2016). Additionally, pathological levels of Aβ peptide block the uptake of glutamate from the synaptic cleft, leading to an increase in its concentration in excitatory synapses (Li et al., 2009). Higher concentrations of extracellular glutamate affect synapses in at least three ways: (1) it may continuously activate NMDA receptors, resulting in the desensitization of these receptors and impairments in LTP (Liu L. et al., 2004); (2) excess glutamate in synapses may cause excitotoxicity that triggers the loss of synapses and dendritic spines; and (3) higher levels of glutamate may activate extrasynaptic NMDA receptors, which, in turn, enhance the induction of LTD (Kollen et al., 2008). Interestingly, patients with the inherited form of AD often suffer from symptoms of epilepsy because of excess glutamate in the synapse in the hippocampus and entorhinal cortex (Palop and Mucke, 2009), further confirming the notion that AD is a synaptopathy. Considering the synaptic deficits that are associated with AD and crucial physiological roles that are played by synaptic metzincins, below we discuss the contributions of different metzincins to AD pathogenesis and treatment.

Higher activity of numerous extracellular proteases and an imbalance between proteases and their inhibitors in the brain play a crucial detrimental role in various pathological conditions (Rosenberg, 2009). Seizures are associated with high levels of MMPs in serum and the CNS. Consequently, the proteolysis of certain extracellular or membrane proteins may affect the pathogenesis of epilepsy. Interestingly, post-mortem analyses of hippocampal tissue from AD patients found elevated levels of several metzincins (Wang X. X. et al., 2014). High expression of MT1-MMP was found in the thalamus, in the smooth muscle cell medial layer of meningeal vessels MMP and in reactive astrocytes in the vicinity of Aβ deposits in aged Tg-SwDI AD mouse brain (Liao and Van Nostrand, 2010). Another study found that human glioma cells (Deb et al., 1999) and human cerebrovascular tissue (Jung et al., 2003) expressed MT1-MMP in response to exposure to pathogenic Aβ peptide. Another member of the MT-MMP family, MT5-MMP, co-localized with senile plaques but was not present in vascular amyloid plaques in post-mortem AD brains (Sekine-Aizawa et al., 2001).

Among MMPs, both membrane and extracellular soluble proteases are upregulated after exposure to Aβ. Higher expression of MMP-2, MMP-3 and MMP-9 was observed in the presence of Aβ (Deb et al., 1999; Jung et al., 2003; Lee et al., 2003). Moreover, MMP-2, MMP-3 and MMP-9 were secreted especially from astrocytes (Figure 1D; Yoshiyama et al., 2000; Deb et al., 2003). MMP-9 was detected in the cytoplasm of neurons and co-localized with neurofibrillary tangles and Aβ plaques (Yoshiyama et al., 2000; Yin et al., 2006; Mizoguchi et al., 2011b). High MMP-3 levels were found in plasma and CSF in AD patients post-mortem (Horstmann et al., 2010). Additionally, intracerebroventricular injections of Aβ25–35, Aβ1–40 and Aβ1–42 increased MMP-9 expression in the hippocampus (Mizoguchi et al., 2009). Pretreatment with the NMDA receptor antagonist MK-801 blocked this effect, indicating the role of NMDA receptors in the Aβ-induced increase in MMP-9 expression (Mizoguchi et al., 2009). Pathological levels of Aβ cause cognitive impairment and dysfunction, but these effects do not occur in MMP-9 deficient mice after an intracerebroventricular injection of Aβ1–40 (Mizoguchi et al., 2009). Interestingly, plasma MMP-9 levels have been reported to either decrease (Horstmann et al., 2010) or increase (Lorenzl et al., 2003) in AD patients. Therefore, despite the large number of tests that have been conducted using different animal models and different biological materials, data on the expression and activity of MMPs in AD patients have been inconsistent. Altogether, the above findings suggest that MMPs are controlled at many levels of their activity in the AD brain. An intriguing issue is whether metzincin upregulation plays a direct role in the amyloidogenic process and synaptic dysfunctions by regulating APP metabolism or degrading Aβ fibrils. If so, then another issue is which specific types of metzincins are key players in AD pathology.

### Amyloid Precursor Protein and its Cleavage

APP is a large transmembrane glycoprotein that is highly expressed in the brain. It is involved in several physiological processes, such as neuronal growth (Young-Pearse et al., 2008; Wang S. et al., 2016), the proliferation and differentiation of neural stem cells and progenitor cells (Hu et al., 2013; Bolós et al., 2014), and synaptic plasticity. Numerous studies have reported that APP promotes the formation of dendritic spines (Lee et al., 2010), alters the expression of NMDA receptors on the cell surface (Hoe et al., 2009; Innocent et al., 2012), and regulates LTP and spatial memory (Taylor et al., 2008). However, the precise roles of APP in these processes under normal conditions remain unknown (Dawkins and Small, 2014).

**Figure 3:**
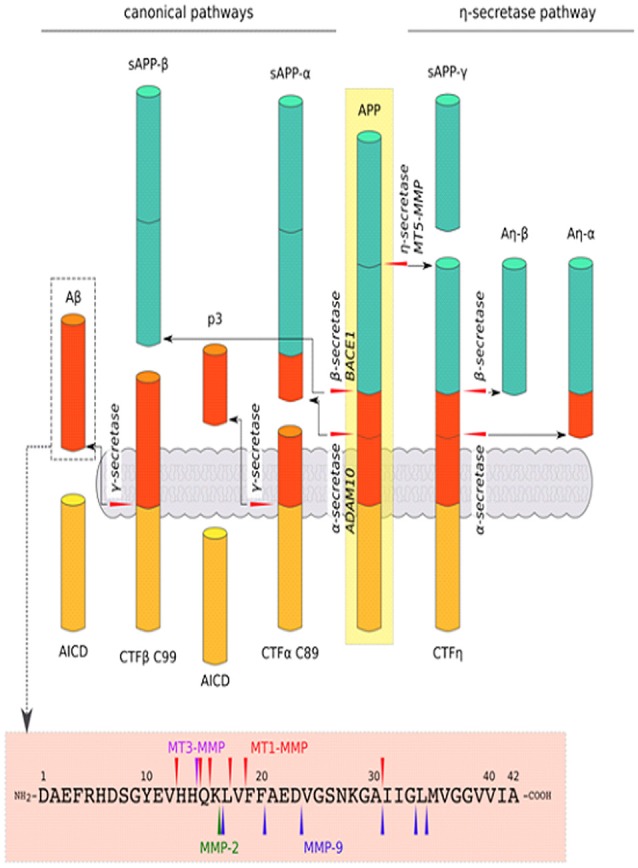
Schematic cleavage of APP in the canonical pathways by α-, β-secretase, a new cleavage pathway by η-secretase and Aβ1–42 degradation by MMPs: MMP-2, MMP-9, MT1-MMP and MT3-MMP. In the canonical pathway, APP is cleaved by β-secretase, generating the secreted ectodomain sAPP-β and membrane-associated C-terminal fragment (CTFβ) C99, which is then cleaved by γ-secretase to yield amyloid β (Aβ) peptide and the APP intracellular domain (AICD; i.e., amyloidogenic pathway). APP can also be cleaved by α-secretase in the canonical pathway. Cleavage by this mechanism results in sAPP-α and CTFα C89, which are further processed by γ-secretase to generate p3 and AICD (i.e., non-amyloidogenic pathway). Another mechanism, via η-secretase in the non-canonical pathway, yield sAPP-η and CTFη, which are further cleaved by α- and β-secretase to Aη-α and Aη-β fragments, respectively. Moreover, various proteinases, especially MMPs, that act beyond APP processing (described above) are involved in the direct degradation of Aβ peptide (O’Brien and Wong, 2011; Dawkins and Small, 2014; Zhang et al., 2015; Andrew et al., 2016; Wang Y. Q. et al., 2016).

The alternative splicing of mRNA results in different APP isoforms. APP695 is expressed predominantly in neuronal cells (Kang et al., 1987). On the cell surface, APP undergoes sequential cleavage by proteases, termed secretases, through several pathways (Figure 3). In the amyloidogenic pathway, APP is first cleaved by β-secretase to yield the soluble extracellular domain of APP (sAPPβ) and membrane-associated C-terminal fragment β (CTFβ) C99, which is then cleaved by γ-secretase, generating Aβ peptide and the APP intracellular domain (AICD). Another mechanism that is mediated by α-secretase is the production of sAPPα and membrane-associated CTFα C89, which is further processed by γ-secretase to yield soluble p3 and AICD. This non-amyloidogenic pathway prevents the generation of Aβ. Although amyloidogenic and non-amyloidogenic pathways have been thoroughly investigated, recent studies showed that not only α-, β- and γ-secretases are involved in APP cleavage (through canonical pathways) but also δ- and η-secretases. The cleavage of APP by δ-secretase generates three soluble fragments and CTFs. CTFδ is a better substrate for β-secretase than full-length APP. Furthermore, η-secretase cleaves APP to a soluble fragment sAPPη and membrane-bound CTFη, which is further cleaved by α- and β-secretases to Aη-α and Aη-β fragments, respectively. The cleavage of APP by η-secretase is 10 times more effective than by β-secretase (O’Brien and Wong, 2011; Dawkins and Small, 2014; Zhang et al., 2015; Andrew et al., 2016; Wang Y. Q. et al., 2016). All of these processes give rise to the production of different peptides and soluble APP domains with diverse synaptic functions (see Figure 3 for more basic details). Not all proteolytic products of APP processing or shedding are neurotoxic. For example, the activity of α-secretase results in the release of sAPPα, which positively affects synaptic plasticity and learning in rodents (Ring et al., 2007; Taylor et al., 2008). However, still unclear are the determinants of the processing pathways that are activated. The crucial factor in this process may be the segregation of APP and respective secretases into lipid rafts or endocytic vesicles (Ehehalt et al., 2003; O’Brien and Wong, 2011).

### Role of Metzincins in APP Cleavage

One of the main causal processes that cause neurodegeneration in AD is the amyloidogenic pathway of APP processing, which leads to the formation of Aβ plaques. Numerous proteases, including adamlysins, MMPs, and astacins, can act as specific secretases and thus regulate extracellular Aβ levels. Therefore, metzincins have emerged as key factors in Aβ metabolism.

Among the ADAM family, ADAM10 (but not ADAM9 or ADAM17) has particularly strong constitutive α-secretase activity *in vivo* (Kuhn et al., 2010). However, the identity of inducible regulated α-secretases is more elusive. MT3-MMP and MT1-MMP have a cleavage site at amino acids H685-Q686 (H14-Q16 of Aβ) and are considered to have α-secretase activity (see Figure 3; Ahmad et al., 2006). MMP-9, which is expressed in an activity-dependent manner, is important for normal synaptic plasticity, but it also has α-secretase activity. This notion is supported by observations that MMP-9-overexpressing transgenic mice have elevated levels of sAPPα, greater LTP in the hippocampus, and enhanced memory (Fragkouli et al., 2012). Furthermore, MMP-9 overexpression in a mouse model of AD reduced the level of Aβ and its oligomers in the brain and restored learning deficits to the levels of wildtype animals (Fragkouli et al., 2014).

β-site APP-cleaving enzyme 1 (BACE1) is an aspartyl protease that has prominent β-secretase activity (Dawkins and Small, 2014). One member of the astacin family, meprin β, may also act as a β-secretase and can compete with ADAM10 in APP cleavage (Schönherr et al., 2016). Moreover, MT5-MMP is considered a β-secretase. MT5-MMP was recently shown to mediate APP processing in a pro-amyloidogenic manner (Baranger et al., 2016, 2017), and this enzyme emerged as an important pathogenic modulator in AD. Baranger et al. (2016) showed that the interaction between MT5-MMP and APP led to the formation of Aβ and the toxic C99 APP fragment. Moreover, MT5-MMP deficiency in 5xFAD transgenic mice (i.e., a model of AD) decreased Aβ load, attenuated gliosis and reversed deficits in learning and LTP (Baranger et al., 2016, 2017). Additionally, a recent study found that MT5-MMP and soluble MMPs contributed to η-secretase activity (Willem et al., 2015). MT5-MMP can cleave at amino acids 504–505 of APP695 to release the CTF-η domain, which has been recently reported to be a novel synaptotoxic fragment that originates from APP (Willem et al., 2015). γ-Secretase has been shown to be a multiprotein complex that comprises four protein subunits: aspartyl protease presenilin 1 or 2, presenilin enhancer 2, nicastrin, and anterior pharynx-defective phenotype (APH-1). The key role of this enzyme is to remove a transmembrane fragment of processed protein (Dawkins and Small, 2014). δ-Secretase is a pH-controlled cysteine protease, termed asparagine endopeptidase (AEP), that cleaves proteins after asparagine residues (Zhang et al., 2014, 2015). Non-MMPs are beyond the scope of the present review; therefore, we refer only to a recent comprehensive review on this topic (Andrew et al., 2016). As noted above, various metzincins work at different sites for APP processing (Figure 3). Unsurprising is that they have attracted increasing attention in the field of therapeutic strategies to regulate Aβ levels and other toxic APP fragments. The augmentation of α-secretase activity (ADAM10, MT3-MMP and MT1-MMP, MMP-9**)** may improve cognitive function and slow the progression of AD. Sirtuins are also well known for their role in extending the longevity of experimental animals. Remarkably, the activity of sirtuins induces the expression of ADAM10, reduces the production of Aβ peptide and amyloid plaques, and improves memory in mice (Theendakara et al., 2013; Lee H. R. et al., 2014).

### Proteolytic Degradation of Aβ by Metzincins

The mechanisms of the proteolytic processing of Aβ and its deposits are of primary interest in the context of AD because such knowledge may reveal future therapeutic strategies. The hallmark of AD is the deposition of Aβ plaques. Amyloid β may also play a beneficial physiological role in the regulation of vesicle release probability in the hippocampus (Abramov et al., 2009) through an unknown mechanism.

Amyloid β occurs most frequently in the form of Aβ1–40 and Aβ1–42 peptides, which comprise 40 and 42 amino acids (4.2 kDa), respectively, and create toxic aggregates that form insoluble plaques or soluble oligomers. Regions between residues 17–21 and 30–35 are critical for Aβ aggregation and responsible for resistance to proteolytic degradation (Liu R. et al., 2004). However, the specific “trimming” of Aβ produces shorter and harmless peptides. Numerous findings indicate that various MMPs that act beyond APP processing are involved in the direct degradation of Aβ and thus may prevent the formation of amyloid fibrils. MT1-MMP was shown to cleave soluble Aβ40 and Aβ42 peptides *in vitro* (Liao and Van Nostrand, 2010). Purified MT1-MMP enzyme without the carboxyl-terminal transmembrane region degraded amyloid plaques in brain tissue (Liao and Van Nostrand, 2010). In contrast to MT1-MMP, MT3-MMP was unable to cleave Aβ peptide (Liao and Van Nostrand, 2010). In addition to MT1-MMP, several other enzymes that are able to degrade the soluble form of Aβ have been identified *in vitro*: MMP2 (Roher et al., 1994), plasmin (Ledesma et al., 2000), endothelin-converting enzyme (ECE; Eckman et al., 2001), insulin-degrading enzyme (IDE; Kurochkin and Goto, 1994), neprilysin (NEP; Howell et al., 1995) and MMP-9 (Backstrom et al., 1996; Yan et al., 2006). All these proteases have specific cleavage sequences in characteristic soluble Aβ40- and Aβ42-generating fragments, although some of these cleavage sites overlap with each other. For example, MT1-MMP can cleave between residues A30 and I31, similar to MMP-9 (Yan et al., 2006; Liao and Van Nostrand, 2010). However, other MT1-MMP cleavage sites were identified in the region between V12 and L17 that are similar to the sequences that are cleaved by IDE (e.g., H14-Q16, Q16-K16; Yan et al., 2006; Liao and Van Nostrand, 2010). MMP-9 can also cleave the soluble form of Aβ into Aβ1–20 and Aβ1–30 fragments that, in turn, attenuate β-pleated sheet formation (Yan et al., 2006; Hernandez-Guillamon et al., 2015). Different studies have used recombinant human MMP-2 (rhMMP-2) and rhMMP-9 and found that these MMPs can generate other fragments, such as Aβ1–34, Aβ1–30 and Aβ1–16 from Aβ1–40 and Aβ1–42. Further digestion was not observed when proteases were incubated with Aβ1–16 (Hernandez-Guillamon et al., 2015). MMP-2 can cleave at residues between K16 and L17 within the hydrophobic domain of Aβ (Miyazaki et al., 1993). Interestingly, APP contains a proteinase inhibitor domain for MMP-2 (Higashi and Miyazaki, 2003).

### Non-Classical Hypotheses of AD Progression: The Role of Metzincins in Neuroinflammation and Aβ Transport in Brain Parenchyma and Across Brain Barriers

Uncontrolled MMP activity may promote CNS pathologies, including BBB and BCSFB disruption, neuroinflammation, and cell apoptosis, and is believed to aggravate neurodegenerative disorders. Indeed, neuroinflammation and enhanced MMP activity have been implicated in the pathogenesis of AD (Wang X. X. et al., 2014). High concentrations of Aβ oligomers and tau protein and neuronal degeneration lead to the activation of immune cells, the release of cytokines and chemokines, and the dysregulation of transduction pathways in the immune response (Heneka et al., 2015). Recent studies found evidence that secreted MMPs enhance AD-related neuroinflammation. For example, MMP-2 and MMP-9 can generate the production of interleukin 1β (IL-1β) and transforming growth factor β (TGFβ) in microglia surrounded by amyloid plaques (Li W. et al., 2011). MMP-9 can also cleave IL-1β, converting it into a more active form (Vandooren et al., 2014).

Growing evidence indicates that studies of the pathomechanisms of neurodegenerative disorders should not focus solely on neurons and glia (i.e., the brain parenchyma); they must also consider interactions with blood vessels and the immune system. For example, several pathways for Aβ clearance mainly via BBBs that contain specific receptors that mediate the uptake of Aβ have been investigated. The tightness of brain barriers appears to be compromised in AD. Experimental studies have indicated a pivotal role for MMPs in the development of such impairments (see “Interplay between Extracellular Proteases and Aβ Receptors LRP1 and receptor for advanced glycation end products (RAGE) in the Regulation of Aβ Levels” Section). Indeed, IL-1β that is activated by MMPs under inflammatory conditions increases BBB permeability (Labus et al., 2014; Wang Y. et al., 2014). Additionally, circulating forms of Aβ initiate the secretion of MMPs from the BBB and BCSFB to CSF (see “Role of MMPs in the Molecular Mechanism of AD Induced by Aβ Oligomers in the Blood-Brain Barrier and Blood-Cerebrospinal Fluid Barrier” Section; Brkic et al., 2015). However, still unknown is the specific role of these proteases in the molecular mechanism of AD symptoms that are induced by Aβ oligomers.

### Central Nervous System Barriers: Blood-Cerebrospinal Fluid Barrier and Blood-Brain Barrier

**Figure 4:**
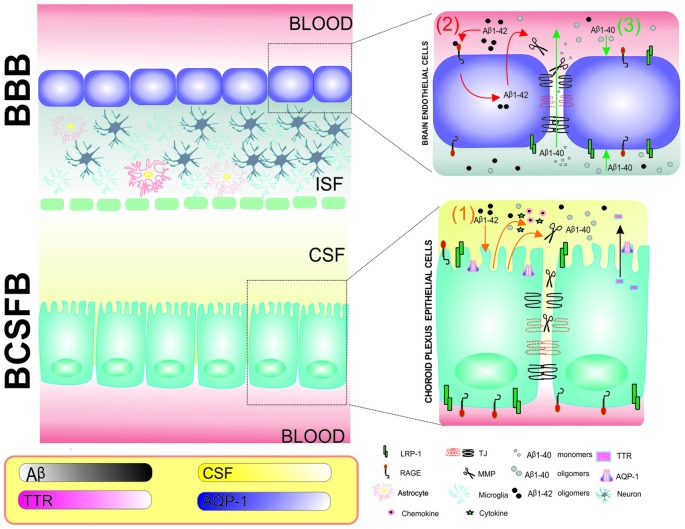
Schematic representation of the involvement of MMPs and effects of Aβ1–40 and Aβ1–42 in the blood-brain barrier (BBB) and BCSFB in Alzheimer’s disease (AD) pathology. Choroid plexus epithelial (CPE) cells and brain endothelial cells (BECs) are tightly connected by tight junctions (TJs) and form BCSFB and BBB monolayers, respectively. (1) An intracerebroventricular injection of Aβ1–42 results in MMP, cytokine and chemokine secretion from CPE cells into CSF. The secreted MMPs further damage TJs at the BCSFB and increase permeability (orange; Brkic et al., 2015). (2) Aβ1–42 interacts with receptor for advanced glycation end products (RAGE) in the BBB, resulting in the expression of MMPs, dysregulation of TJs and breakdown of the BBB (red; Kook et al., 2012). (3) Keaney et al. (2015) showed that Aβ1–40 peptide leads to lower expression of TJ proteins and allows Aβ40 monomers to diffuse from brain to blood (green). The frame describes changes in CPE cell function that are associated with AD, including the lower production of CSF, lower expression of AQP-1 in CPE cells and lower concentrations of transthretin (TTR) in CSF in the 3xTg-AD mouse model (González-Marrero et al., 2015).

**Figure 5:**
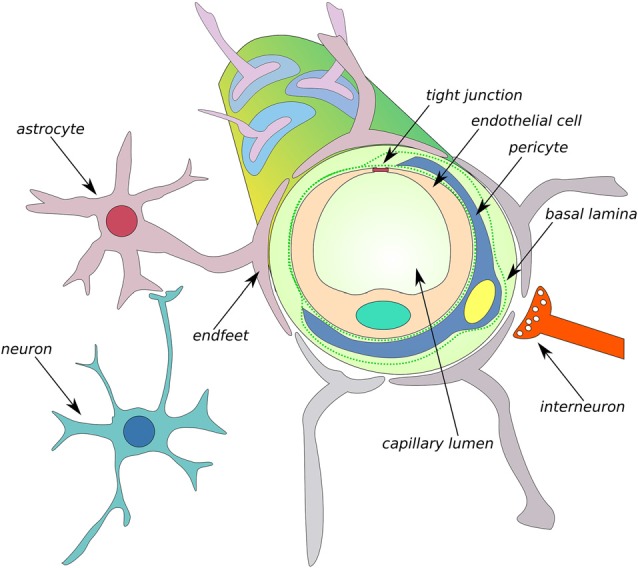
Schematic of the neurovascular unit (NVU) of the BBB. The BBB is formed by endothelial cells that are interconnected by tight junction (TJ) connections and neighboring cells, such as astrocytes, microglia, interneurons, neurons, and pericytes, thus comprising the NVU. MMPs can cleave TJs and degrade the composition of basal lamina, such as collagen IV, elastin, proteoglycans, laminin and fibronectin, leading to an increase in BBB permeability (Abbott et al., 2006).

In addition to aberrant processes of Aβ production in the brain, an imbalance in Aβ clearance may also lead to the progression of AD. Before we discuss the key role of metzincins in the modulation of Aβ transport and clearance, we briefly describe the functioning of the main CNS barriers under physiological and pathological conditions. The BBB, BCSFB, and arachnoid barrier are dynamic complexes that maintain brain homeostasis, detect changes in CSF or blood, and provide balanced microenvironments that are critical for protecting the CNS from damage (Abbott et al., 2010; Redzic, 2011; de Wit et al., 2016). The BBB and BCSFB have the largest surface interface between blood and brain extracellular fluids. The BBB separates brain parenchyma and interstitial fluid from the blood. The BCSFB is a barrier between capillaries and CSF (Abbott et al., 2010). The molecular biology of the BBB and BCSFB has both similarities and differences. For example, the BCSFB is a monolayer that is composed of choroid plexus epithelial (CPE) cells, whereas the BBB is composed of brain endothelial cells (BECs; Figure 4). The main function of CPE cells is the production of CSF. Both CPE cells and BECs are interconnected with such structural components as tight junctions (TJs) and adherens junctions (AJs). TJs consist of transmembrane and membrane-associated cytoplasmic proteins, such as claudins and occludins, that interact with actin filaments and trigger intracellular signaling. Because of the anatomical localization of the BBB, this layer is surrounded by astrocytes, microglia, interneurons, excitatory neurons, and pericytes and interacts with circulating immune cells (Figure 5). Altogether, the BBB and its neighboring cells are defined as the neurovascular unit (NVU; Abbott et al., 2006).

Various approaches have been used to study crosstalk between cells in the NVU. For example, mice that lacked the water channel aquaporin 4 (AQP-4) in astrocyte endfeet were characterized by lower CSF flux and the elimination of interstitial solute clearance (Iliff et al., 2012). Both layers, the BSCFB and BBB, regulate the paracellular pathway to move electrolytes between brain extracellular fluids and blood, transport important molecules (e.g., glucose and amino acids) from the blood, and clear metabolic waste to the blood. BEC and CPE cells express many specific transporters and receptors that allow them to maintain these functions (see “Interplay between Extracellular Proteases and Aβ Receptors LRP1 and RAGE in the Regulation of Aβ Levels” Section).

Increases in Aβ levels in the CNS and periphery result in BBB and BCSFB dysfunction (Kook et al., 2012; Brkic et al., 2015). A new neurovascular hypothesis of AD postulates that deficits in the elimination of Aβ across the BBB results from impairments in Aβ transporters that are located in brain capillaries (Storck et al., 2016). The mechanisms by which Aβ oligomers affect the CPE-CSF system and functions of CPE cells and BECs in the progression of AD await elucidation. However, some light on this problem has been shed by model studies that are based on the adult triple transgenic AD mouse (3xTg-AD) that harbors three human mutant genes: β-APP (APPSwe), PS1/M146V, tauP301L. In the 3xTg-AD model, the transport and metabolic function of CPE cells was compromised, giving rise to impairments in CSF production, CSF flow, and Aβ clearance (González-Marrero et al., 2015). The expression of AQP-1 in CPE cells and concentration of transthyretin (TTR) in CSF were reduced in 3xTg-AD mice compared with wildtype mice (Figure 4). Consistent with the well-established role of TTR in preventing amyloid plaque formation, many studies confirmed that AD patients had lower concentrations of this protein in CSF (Serot et al., 1997). Moreover, TTR was shown to be produced in neurons in a mouse model that overexpressed human APP695 (Tg2576; Stein and Johnson, 2002). Some aspects of AD pathology can also be reproduced by injecting Aβ1–40 or Aβ1–42 oligomers into the cerebral ventricles, which disrupts the BBB and BCSFB (Kook et al., 2012; Brkic et al., 2015). CPE cell and BEC dysfunction is also associated with the release of proinflammatory molecules into CSF (Brkic et al., 2015). In summary, changes in CPE cell function are important for cell-to-cell communication and Aβ clearance. However, still unknown are the mechanisms that underlie the uptake of Aβ oligomers from CSF by CPE cells.

### Interplay between Extracellular Proteases and Aβ Receptors LRP1 and RAGE in the Regulation of Aβ Levels

Recent studies suggest that higher Aβ levels in the brain do not result from elevated production but rather from impaired clearance mechanisms in the CNS. Interestingly, impairments in Aβ transport across the BBB also results in learning deficits. The major mechanisms that are involved in maintaining Aβ at low physiological levels include elimination via interstitial fluid bulk flow, removal into the circulatory and lymphatic systems through CSF, degradation in tissue and transvascular transport across the BBB, which is supposed to be the major route of Aβ elimination. Different receptors are involved in the latter mechanism, but a particularly prominent role is played by LRP1 and RAGE (Mawuenyega et al., 2010; Tarasoff-Conway et al., 2015; Storck et al., 2016).

RAGE is a 55 kDa protein that consists of three structural regions: a cytoplasmic tail that conducts the signal to the cytoplasm, a transmembrane domain and an extracellular region that binds the ligands. RAGE has numerous ligands, such as advanced glycation end products (AGEs), Aβ, high mobility group box 1 (HMGB-1) and S100 calcium-binding protein B (S100B). Therefore, RAGE may play an important role in both physiological and pathological brain processes. The aberrant expression of RAGE and its ligands occurs in some disorders, especially AD, which may lead to the development of neuroinflammation (Schmidt et al., 1992, 1994; Yan et al., 1994; Chuah et al., 2013; Piras et al., 2016).

LRP1 is a large 600 kDa protein that is representative of the low-density lipoprotein receptor family. Under physiological conditions, it can bind to the cell surface through the transmembrane domain or be present in its soluble form (sLRP1; Ramanathan et al., 2015). In addition to binding Aβ, LRP1 binds various ligands, such as apolipoprotein E (apoE), tPA, and APP, among many others (Zlokovic et al., 2010). Interestingly, LRP1 mediates the transport of Aβ from the brain to the blood and is primarily expressed on the abluminal side of the BBB (Zlokovic et al., 2010). It also plays an important role in lipoprotein metabolism and synaptic transmission in the CNS (Bu, 2009). Treatment with receptor-associated protein, a scavenger that interferes with ligand-LRP1 interactions, reduced the late-phase of LTP (Zhuo et al., 2000). Moreover, the binding of tPA to LRP1 upregulated cyclic adenosine monophosphate-dependent protein kinase, which regulates hippocampal LTP (Zhuo et al., 2000). The lack of LRP1 in primary neurons decreased the levels of glutamate A1 (GluA1) subunits, reduced the phosphorylation of GluA1 at serine 845 and serine 831, attenuated calcium influx through NMDA receptors and led to deficits in LTP (Gan et al., 2014).

LRP1 and RAGE transport Aβ in opposite directions. RAGE is responsible for Aβ transport through the BBB from the blood to the brain (Pascale et al., 2011), whereas LRP1 transports Aβ in the opposite direction. González-Marrero et al. (2015) found that the same mechanism might be involved in the BCSFB because both LRP1 and RAGE are expressed by CPE cells (González-Marrero et al., 2015). The cytoplasmic expression of LRP1 and RAGE in CPE cells in 3xTg-AD mice increased compared with wildtype animals (González-Marrero et al., 2015). However, lower LRP1 levels were observed in AD brains (Kang et al., 1987). Elucidation of the specific roles of RAGE and LRP1 in Aβ clearance via the BBB has been difficult because of the lack of appropriate animal models. For example, global knockout of the *Lrp1* gene in mice was embryonically lethal (Herz et al., 1992, 1993). Several studies have utilized specific antibodies or antagonists that block the ligand binding site (Shibata et al., 2000; Yamada et al., 2008). Alternatively, gene deletion that is limited to specific cell types can be used. For example, the selective deletion of *Lrp1* in BECs in mice reduced Aβ in plasma and increased soluble Aβ in the brain, indicating a decrease in Aβ efflux from the brain (Storck et al., 2016). Notably, there are differences in the rate of Aβ clearance between mice and humans, with five-fold higher clearance in the mouse brain (Qosa et al., 2014).

Several proteases can cleave extracellular regions of RAGE and LRP1, creating their soluble forms that are still able to bind the ligand, mainly Aβ. MMP-9 and ADAM10 are able to cleave RAGE (Zhang et al., 2008; Chuah et al., 2013). In turn, many members of the ADAM family, such as ADAM-10 and ADAM-17, are engaged in the shedding of LRP1 (Liu et al., 2009). Freely circulating soluble forms of both receptors bind peripheral Aβ and thus preclude the transport of unbound Aβ into the brain. Consequently, the activity of MMPs in the blood diminishes Aβ loading in the brain. Under normal conditions, the rates of fractional synthesis and clearance of Aβ in CSF are estimated to be 7.6% and 8.3% per hour, respectively (Bateman et al., 2006). More than 70% of circulating Aβ normally binds to a soluble form of LRP1 (sLRP1). Oxidized sLRP1 in AD has lower affinity for Aβ, resulting in an increase in free Aβ1–40 and Aβ1–42 in plasma and enhanced Aβ transport into the brain through RAGE (Sagare et al., 2007, 2012). Interestingly, the inhibition of ADAM proteases (e.g., ADAM-10) resulted in a reduction of LRP1 shedding at the BEC surface and increased Aβ1–42 transport by RAGE through the BBB to the brain (Sagare et al., 2007; Shackleton et al., 2016).

### Role of MMPs in the Molecular Mechanism of AD Induced by Aβ Oligomers in the Blood-Brain Barrier and Blood-Cerebrospinal Fluid Barrier

As described above, the structures of the BBB and BCSFB are very complex, and their functions depend on several molecular players. Among the factors that are known to strongly affect the functioning of these barriers are extracellular proteases. MMPs are known to digest the composition of the basement membrane, which mostly includes a variety of classes of ECM molecules, such as collagen type 4, elastin, laminin, fibronectin and proteoglycans. This degradation of the basal lamina impairs ECM function and leads to the disruption of TJs between BECs and BBB breakdown (Carvey et al., 2009). The soluble forms of Aβ42 were shown to interact with RAGE and initiate intracellular cascades that resulted in the secretion of MMPs, such as MMP-2 and MMP-9, in BBB BEC cultures (Kook et al., 2012; Figure 4).

Aβ40 monomers and dimers decreased the expression of claudin-5 and occludin in BECs *in vitro* (Keaney et al., 2015). Lower expression of TJ proteins by siRNAs allowed the diffusion of Aβ peptides and oligomers with a molecular mass <8 kDa from brain to blood (Keaney et al., 2015). On the other hand, it was still unclear, whether Aβ oligomers can affect TJ connections in CPE cells. To address this issue, Brkic et al. (2015) intracerebroventricularly injected Aβ42 in mice and found that circulating Aβ42 in CSF induced the expression of several inflammatory molecules (e.g., cytokines and chemokines) and MMPs in CPE cells, resulting in BSCFB leakage.

## Future Perspectives and Conclusions

The emerging picture is that the involvement of extracellular and membrane proteases is a universal feature of long-term synaptic plasticity and learning, and the involvement of these enzymes in physiological and pathological conditions is well known. Various proteases play different neuron- or synapse-specific roles in plasticity and learning by processing or degrading numerous membrane and ECM proteins. Thus, the synapse itself and its perisynaptic microenvironment are the location of complex and highly coordinated proteolytic events that modulate numerous signaling cascades. Unraveling the ways in which different extracellular proteases contribute to the regulation of synaptic plasticity and learning constitutes a major challenge that is currently being investigated at specific synapses (e.g., inhibitory and excitatory) using various behavioral paradigms and in several brain pathologies. A basic unresolved issue is the way in which the synaptic activity of metzincins is activated, maintained and terminated. Bridging the gap between our current knowledge about synaptic dysfunctions in AD and the compromised functioning of extracellular proteolysis (different MMPs and ADAMs in particular) is essential to better understand the pathogenesis of AD and other neurological disorders.

The last few years have seen growing interest in the role of Aβ oligomers in the function of the BCSFB and BBB in the context of AD progression. MMPs play an important role in interactions between Aβ transporters and intracellular signaling that disrupt TJs in CPE cells and BECs. MMPs are also a major factor that cause disintegration of the BBB and BCSFB. Regulating MMP activity may be an important step toward identifying strategies to ameliorate or cure AD. MMPs may be a particularly suitable and efficient target for pharmacological treatment because it is not normally expressed in CPE cells and is specifically recruited following Aβ exposure (Brkic et al., 2015). Thus, MMPs may become a CSF marker of AD pathology. Equally important, MMPs may be upstream of a complex cascade that mediates the pathogenesis of AD. The role of MMP-3 in AD awaits investigation. This protease liberates latent proteins that are critical for the progression of AD (e.g., IL-1β and TNFα) and cleaves adherens junction proteins (e.g., E-cadherins) in brain barriers (Van Hove et al., 2012a). A recent study identified a key role for MMP-3 in promoting blood-spinal cord barrier disruption and hemorrhage and suggested that MMP-3 may be a therapeutic target for neurological recovery after spinal cord injury (Lee J. Y. et al., 2014). Moreover, MMP-3 regulates the availability of MMP-9 and MMP-13, and the former degrades not only soluble but also fibrillary Aβ1–42 (Yan et al., 2006; Van Hove et al., 2012a).

The crucial role of metzincins in several brain diseases has made them attractive therapeutic targets. Unlike the extensively studied gelatinases MMP-2/9, the relative lack of highly specific reagents and investigative tools limits a further understanding of the role of other members of the metzincin superfamily in brain function (Huntley, 2012). Thus, clinical investigations have been limited by the lack of MMP subtype-specific inhibitors (Vandenbroucke and Libert, 2014). Additionally, despite the classic view that MMPs are secreted or membrane proteases, numerous studies have identified several intracellular and nuclear substrates of neuronal MMP-3, MT1-MMP and MMP-9 (Cauwe and Opdenakker, 2010; Choi et al., 2011; Wiera et al., 2012), thus making their brain function more complex and the effective targeting of specific types of MMPs more difficult. In the normal adult brain, MMP activity is indispensable for activity-dependent synaptic plasticity, the long-term scaling of neuronal excitability (Nagy et al., 2006; Bozdagi et al., 2007; Wójtowicz and Mozrzymas, 2014; Wiera and Mozrzymas, 2015; Wójtowicz et al., 2015; Lebida and Mozrzymas, 2016; Brzdak et al., 2017) and learning (Wright and Harding, 2009). Fine-tuned MMP activity differentially affects brain function. Designing pharmacological compounds should consider yet-unknown side effects with regard to cognitive function. Interestingly, AD patients exhibit higher levels of endogenous TIMPs (Lorenzl et al., 2003), which may reflect a natural defense mechanism against higher MMP activity in AD pathogenesis. Therefore, targeting TIMPs may additionally help ameliorate AD progression. However, therapeutic strategies for manipulating MMP activity should ideally maintain an optimal balance between MMPs and TIMPs to avoid complications that result from excess or deficient MMP activity.

## Author Contributions

PB, DN and GW equally contributed to this article. JWM: contribution to the article concept, writing part of the manuscript, supervision and editing.

## Conflict of Interest Statement

The authors declare that the research was conducted in the absence of any commercial or financial relationships that could be construed as a potential conflict of interest.

## Abbreviations

3xTg-AD: triple transgenic AD mouse
ABP: AMPA receptor binding protein
AD: Alzheimer’s disease
ADAMs: A Disintegrin And Metalloproteinases
ADAMTSs: A Disintegrin And Metalloproteinase with Thrombospondin Motifs
AEP: asparagine endopeptidase
AGEs: advanced glycation end products
AICD: APP intracellular domain
AJs: adherens junctions
AMPA: α-amino-3-hydroxy-5-methyl-4-isoxazolepropionic acid
APH-1: anterior pharynx-defective phenotype
apoE: apolipoprotein E
APP: amyloid precursor protein
AQP-4: aquaporin 4
Aβ: amyloid β
BACE1: β-site APP-cleaving enzyme 1
BBB: blood-brain barrier
BCSFB: blood-cerebrospinal fluid barrier
BDNF: brain-derived neurotrophic factor
BEC: brain endothelial cell
BMP1: bone morphogenetic protein-1
CaMKII: Ca^2+^/calmodulin-dependent protein kinase
CNS: central nervous system
CPE: choroid plexus epithelial
CTF: membrane-associated C-terminal fragment
ECE: endothelin-converting enzyme
ECM: extracellular matrix
GluA1: glutamate A1
GPI: glycosylphosphatidylinositol
GRIP: glutamate receptor interacting protein
HMGB-1: high mobility group box 1
ICAM-5: intercellular adhesion molecule 5
IDE: insulin-degrading enzyme
IGF: insulin-like growth factor
IL-1β: interleukin 1 β
LRP1: lipoprotein receptor-related protein 1
LTD: long-term depression
LTP: long-term potentiation
MMPs: metalloproteinases
NEP: neprilysin
NMDA: *N*-methyl-D-aspartate
NPTX: neuronal pentraxin
NVU: neurovascular unit
PAR-1: Protease-activated receptor-1
RAGE: receptor for advanced glycation end products
rhMMP: recombinant human MMP
S100B: S100 calcium-binding protein B
SAP97: synapse-associated protein 97
sAPP: soluble extracellular domain of APP
sLRP1: soluble form of LRP1
TGFβ: transforming growth factor β
TIMP-1: tissue inhibitor of metalloproteinases-1
TJs: tight junctions
Tll: tolloid-like
TNF-α: tumor necrosis factor α
tPA: tissue plasminogen activator
TrkB: tropomyosin receptor kinase B
TTR: transthyretin.
