# Supplementary material for: Multifaceted Roles of Metzincins in CNS Physiology and Pathology: From Synaptic Plasticity and Cognition to Neurodegenerative Disorders
Source: Front Cell Neurosci. 2017 Jun 30;11:178. doi: 10.3389/fncel.2017.00178 (PMC5491558; doi:10.3389/fncel.2017.00178)
Supplement: Supplementary file 1 [file Table_1.pdf]

MMP Protease	mRNA expression in mouse brain (http://mouse.brain-map.org/)	mRNA expression in human cerebral cortex (The Human Protein Atlas)	Protein expression in human cerebral cortex (The Human Protein Atlas)	Comments and literature
MMP-1 (collagenase 1)	---†	---	ND	- Detected at low level in rodent cortex; upregulated by kainate (Ierusalimsky and Balaban, 2013)<br>- Present at low level in human cortex (Leake et al., 2000)
MMP-2 (gelatinase A)	---†	+	+<br>(mainly neurons)	- In rat hippocampus expression is the highest during the first postnatal week but present also in adult brain (Aujla and Huntley, 2014);<br>- Reviewed in Verslegers et al. (2013)
MMP-3 (stromelysin 1)	---†	---	+++ #<br>(neurons, astrocytes and neuropil)	- Punctate expression in hippocampal slices, mainly in astrocytes (Wiera et al., 2017)<br>- Expressed by Purkinje cells and Golgi cells in postnatal cerebellum (Van Hove et al., 2012b);<br>- reviewed in Van Hove et al. (2012a)
MMP-7 (matrilysin)	---†	---	+#	- High expression in neurons in the hippocampus under basal conditions (Le and Friedman, 2012)
MMP-8 (collagenase 2)	---†	---	---	- Below the level of detection in brain homogenates; but present in microglia after ischemia or neuroinflammation (Lee et al., 2014; Han et al., 2016)
MMP-9 (gelatinase B)	+†	---	---	- Expressed in the rodent brain in activity-dependent manner; reviewed in Dziembowska and Wlodarczyk (2012); Verslegers et al. (2013); Kaczmarek (2016)
MMP-10 (stromelysin 2)	---†	---	ND	- Present in striatal cells, where it directly cleaves huntingtin and prevents cell death (Miller et al., 2010)
MMP-11 (stromelysin 3)	---†	---	---	

MMP-12 (macrophage elastase)	---†	---	ND	- Produced by microglia and oligodendrocytes in vitro (Larsen and Yong, 2004); upregulated in vivo after axonal degeneration (Liao et al., 2015), neuroinflammation in the aged brain (Liu et al., 2013) and ischemia (Chelluboina et al., 2015)
MMP-13 (collagenase 3)	+++†	---	ND	- Expressed at low level in mammalian brain, upregulated after ischemia (Cuadrado et al., 2009; Lu et al., 2009; Ma et al., 2016)
MMP-14 (MT1-MMP)	---	+	+ (neuropil and neurons)	- Upregulated after BDNF treatment in neuronal cultures (Cazzin et al., 2011)<br>- Punctate distribution in hippocampal stratum pyramidale (Py et al., 2014)
MMP-15 (MT2-MMP)	+++	+	+++ (mainly neuropil)	- High level in oligodendrocytes in vitro and in optic nerve (Sharma et al., 2015)
MMP-16 (MT3-MMP)	+++	++	++# (neurons and astrocytes)	- Present in cortical neurons (Ferraro et al., 2011) as well as in astrocytes (Yoshiyama et al., 1998)
MMP-17 (MT4-MMP)	+++†	+++	ND	- High level of neuronal expression in cortex, hippocampus, substantia nigra (Rikimaru et al., 2007), cerebellum and striatum (Sharma et al., 2015)
MMP-19	---†	---	+#	- Expressed in microglia (van Horssen et al., 2006)
MMP-20 (enamelysin)	---†	ND	ND	
MMP-21	---†	---	++ #	- Expressed in embryonal neocortex (Marchenko et al., 2003)
MMP-23B	---†	---	+#	- Not detected in brain homogenates (Velasco et al., 1999)
MMP-24 (MT5-MMP)	+†	+	+++# (mainly neurons)	- Expression in the hippocampus, dentate gyrus, olfactory bulb (Jaworski, 2000) and cerebellum (Sekine-Aizawa et al., 2001)<br>- Neuronal expression (Sharma et al., 2015), present also in dendritic spines (Monea et al., 2006)

MMP-25 (MT6-MMP)	+†	---	+++#	- Expressed in fetal rat brain (Bernal et al., 2005)
MMP-26 (matrilysin-2)	Only human protease	---	ND	
MMP-27	--- †	---	---#	
MMP-28 (epilysin)	---†	ND	ND	- mRNA not detected in rodent adult cortex and cerebellum (Bernal et al., 2005)<br>- Expressed in mice in the developing and regenerating nervous system prior to myelination (Werner et al., 2007)

Table S1. Expression of different MMPs in mouse and human brain in non-pathological conditions. Only proteases present in human genome are considered. Pseudogene MMP23A is omitted. # indicates uncertain conclusion based on staining with single antibody, lack of # mark indicates testing with two or more different antibodies. ND – not determined. † indicates only single inconclusive experiment. When protein expression does not correspond to the presence of mRNA, the used antibody probably shows off-target specificity.

MMP Protease	mRNA expression in mouse brain (http://mouse.brain-map.org/)	mRNA expression in human cerebral cortex (The Human Protein Atlas)	Protein expression in human cerebral cortex (The Human Protein Atlas)	Comments and literature
ADAMTS1	---	-	-[#]	- Expressed in mouse and rat brain during development (Gunther et al., 2005)<br>- mRNA present in adult mice hippocampus (Levy et al., 2015)<br>- Increased expression in cortical somatostatin-positive interneurons after sensory experience (Mardinly et al., 2016)
ADAMTS2	+	+	+++	- Protein detected in cholecystokinine-positive cortical interneurons (Doyle et al., 2008)
ADAMTS3	---[†]	+	+++[#]	- Protein detected in rodent neonatal cerebellum (Sharma et al., 2015)
ADAMTS4	+++	+	+++[#]	- mRNA present in adult mice hippocampus (Levy et al., 2015) in neurons, astrocytes and oligodendrocytes (Sharma et al., 2015)
ADAMTS5	---[†]	---	+++[#]	- mRNA present in adult mice hippocampus (Levy et al., 2015) in neurons, astrocytes and oligodendrocytes (Sharma et al., 2015)
ADAMTS6	---[†]	---	---[#]	
ADAMTS7	---[†]	---	---	
ADAMTS8	---	+	+[#]	- Expressed specifically in PV positive interneurons (Rossier et al., 2015; Mardinly et al., 2016) and in cerebellar Purkinje cells (Dunn et al., 2006)<br>- Increased expression in cortical PV interneurons after learning-related neuronal activity (Mardinly et al., 2016)

ADAMTS9	++†	+	+	- Present in developing mouse cerebral cortex (Jungers et al., 2005)
ADAMTS10	---†	+	+++#	- Expressed in the developing and adult brain (Somerville et al., 2004)
ADAMTS12	+†	+	+++#	
ADAMTS13	---†	+	++	- mRNA present in homogenates of mice brain (Cal et al., 2002; Wang et al., 2012)<br>- Expressed in astrocytes and microglia in vitro (Tauchi et al., 2012)
ADAMTS14	---†	+	+#	- mRNA present in homogenates of mice brain (Cal et al., 2002)
ADAMTS15	---	+	ND	- Expressed specifically in PV positive interneurons (Levy et al., 2015; Rossier et al., 2015; Mardinly et al., 2016)<br>- Present also in astrocytes (Sharma et al., 2015)
ADAMTS16	---	---	ND	- mRNA present in homogenates of mice brain (Cal et al., 2002)
ADAMTS17	+	+	ND	- Lack of mRNA in homogenates of mice brain (Cal et al., 2002)
ADAMTS18	---	+	+++#	- mRNA present in homogenates of mice brain (Cal et al., 2002; Wang et al., 2012)
ADAMTS19	+	---	ND	- Expressed in activity-dependent manner in cortex after sensory experience (Mardinly et al., 2016)
ADAMTS20	---†	ND	++	- Temporal brain expression during development (Rao et al., 2003)

Table S2. Expression of different ADAMTSs in mouse and human brain. Only proteases present in human genome are considered. # indicates uncertain conclusion based on staining with single antibody, lack of # mark indicates testing with two or more different antibodies. ND – not determined. † indicates only single inconclusive experiment. When protein expression does not correspond to the presence of mRNA, the used antibody probably shows off-target specificity.

MMP Protease	mRNA expression in mouse brain (http://mouse.brain-map.org/)	mRNA expression in human cerebral cortex (The Human Protein Atlas)	Protein expression in human cerebral cortex (The Human Protein Atlas)	Comments and literature
ADAM8 (CD 156)	---	+	+#	- Protein present in neurons and oligodendrocytes (Schlomann et al., 2000)<br>- Present in astrocytes and oligodendrocytes in vitro (Sharma et al., 2015)
ADAM9	---†	+++	+++ #<br>(neurons, astrocytes and neuropil)	- mRNA expressed in the hippocampus hypothalamus and in Purkinje neurons (Weskamp et al., 2002)
ADAM10	++†	+	+#	- Expressed in neurons in cerebral cortex, hippocampus and cerebellum (Marcinkiewicz and Seidah, 2000), present in dendritic spines (Malinverno et al., 2010)<br>- Reviewed in (Saftig and Lichtenthaler, 2015)
ADAM12	---†	+	+	- Expressed mainly in oligodendrocytes (Bernstein et al., 2004; Lendeckel et al., 2015)
ADAM15	+++†	+	+++ #<br>(neurons, astrocytes and neuropil)	- High expression in the whole brain (Ortiz et al., 2005)
ADAM17 (TACE)	---†	+++	+++<br>(neuropil and neurons)	- Expressed in the whole brain, especially in neurons (Moro et al., 2003), also by oligodendrocytes in vitro (Palazuelos et al., 2014)
ADAM19	---	+	+#<br>(mainly neurons)	- Widely expressed in nearly every tissue examined (reviewed in Qi et al. (2009))<br>- Increased expression in cortical neurons after sensory experience (Mardinly et al., 2016)
ADAM20	Only human protease	---	---#	- Not expressed in the CNS (Hooft van Huijsduijnen, 1998)

ADAM21	---†	---	---#	- Protein detected in the rodent subventricular zone (Yang et al., 2005)
ADAM28	---†	+	ND	- Not detected in the CNS (Oh et al., 2005)
ADAM30	---†	---	---#	- Expressed in neuron in human brain (Letronne et al., 2016)
ADAM33	---	+	+++#	- Expressed in the brain (Yoshinaka et al., 2002) e.g. in cortex, hippocampus and cerebellum (Gunn et al., 2002; Umland et al., 2003)
meprin α -subunit	---†	---	+	
meprin β -subunit	---†	---	---#	- Present and active in the CNS (Sterchi et al., 2008; Jefferson et al., 2011)
procollagen C-protease (Bone morphogenetic protein 1 , BMP1)	---†	+	+++#	
mammalian tolloid-like 1 protein	---	+	ND	- Abundant expression in cerebellum; present in restricted number of cells in hippocampus (Tamura et al., 2005; Sharma et al., 2015)
mammalian tolloid-like 2 protein	+†	+	ND	- mRNA detected in developing brain (Scott et al., 1999)
Astacin-like metallo-endopeptidase (ovastacin)	---†	---	+++#	
Leishmanolysin Like Peptidase	---†	+	+	- Expressed in spinal cord and striatum (Doyle et al., 2008)

Table S3. Expression of different adamlysins, astacins and leishmanolysin in mouse and human brain. Only proteases present in human genome and with proteolytic activity are considered. # indicates uncertain conclusion based on staining with single antibody, lack of # mark indicates testing with two or more different antibodies. ND – not determined. † indicates only single inconclusive experiment. When protein expression does not correspond to the presence of mRNA, the used antibody probably shows off-target specificity.

Literature

Aujla PK, Huntley GW (2014) Early postnatal expression and localization of matrix metalloproteinases-2 and -9 during establishment of rat hippocampal synaptic circuitry. J Comp Neurol 522:1249-1263.

Bernal F, Hartung HP, Kieseier BC (2005) Tissue mRNA expression in rat of newly described matrix metalloproteinases. Biological research 38:267-271.

Bernstein HG, Keilhoff G, Bukowska A, Ziegeler A, Funke S, Dobrowolny H, Kanakis D, Bogerts B, Lendeckel U (2004) ADAM (a disintegrin and metalloprotease) 12 is expressed in rat and human brain and localized to oligodendrocytes. J Neurosci Res 75:353-360.

Cal S, Obaya AJ, Llamazares M, Garabaya C, Quesada V, Lopez-Otin C (2002) Cloning, expression analysis, and structural characterization of seven novel human ADAMTSs, a family of metalloproteinases with disintegrin and thrombospondin-1 domains. Gene 283:49-62.

Cazzin C, Mion S, Caldara F, Rimland JM, Domenici E (2011) Microarray analysis of cultured rat hippocampal neurons treated with brain derived neurotrophic factor. Mol Biol Rep 38:983-990.

Chelluboina B, Warhekar A, Dillard M, Klopfenstein JD, Pinson DM, Wang DZ, Veeravalli KK (2015) Post-transcriptional inactivation of matrix metalloproteinase-12 after focal cerebral ischemia attenuates brain damage. Sci Rep 5:9504.

Cuadrado E, Rosell A, Borrell-Pages M, Garcia-Bonilla L, Hernandez-Guillamon M, Ortega-Aznar A, Montaner J (2009) Matrix metalloproteinase-13 is activated and is found in the nucleus of neural cells after cerebral ischemia. J Cereb Blood Flow Metab 29:398-410.

Doyle JP, Dougherty JD, Heiman M, Schmidt EF, Stevens TR, Ma G, Bupp S, Shrestha P, Shah RD, Doughty ML, Gong S, Greengard P, Heintz N (2008) Application of a translational profiling approach for the comparative analysis of CNS cell types. Cell 135:749-762.

Dunn JR, Reed JE, du Plessis DG, Shaw EJ, Reeves P, Gee AL, Warnke P, Walker C (2006) Expression of ADAMTS-8, a secreted protease with antiangiogenic properties, is downregulated in brain tumours. Br J Cancer 94:1186-1193.

Dziembowska M, Wlodarczyk J (2012) MMP9: a novel function in synaptic plasticity. Int J Biochem Cell Biol 44:709-713.

Ferraro GB, Morrison CJ, Overall CM, Strittmatter SM, Fournier AE (2011) Membrane-type matrix metalloproteinase-3 regulates neuronal responsiveness to myelin through Nogo-66 receptor 1 cleavage. J Biol Chem 286:31418-31424.

Gunn TM, Azarani A, Kim PH, Hyman RW, Davis RW, Barsh GS (2002) Identification and preliminary characterization of mouse Adam33. BMC genetics 3:2.

Gunther W, Skaftnesmo KO, Arnold H, Bjerkvig R, Terzis AJ (2005) Distribution patterns of the anti-angiogenic protein ADAMTS-1 during rat development. Acta Histochem 107:121-131.

Han JE, Lee EJ, Moon E, Ryu JH, Choi JW, Kim HS (2016) Matrix Metalloproteinase-8 is a Novel Pathogenetic Factor in Focal Cerebral Ischemia. Mol Neurobiol 53:231-239.

Hooft van Huijsduijnen R (1998) ADAM 20 and 21; two novel human testis-specific membrane metalloproteases with similarity to fertilin-alpha. Gene 206:273-282.

Ierusalimsky VN, Balaban PM (2013) Type 1 metalloproteinase is selectively expressed in adult rat brain and can be rapidly up-regulated by kainate. Acta Histochem 115:816-826.

Jaworski DM (2000) Developmental regulation of membrane type-5 matrix metalloproteinase (MT5-MMP) expression in the rat nervous system. Brain Res 860:174-177.

Jefferson T, Causevic M, auf dem Keller U, Schilling O, Isbert S, Geyer R, Maier W, Tschickardt S, Jumpertz T, Weggen S, Bond JS, Overall CM, Pietrzik CU, Becker-Pauly C (2011) Metalloprotease meprin beta generates nontoxic N-terminal amyloid precursor protein fragments in vivo. J Biol Chem 286:27741-27750.

Jungers KA, Le Goff C, Somerville RP, Apte SS (2005) Adamts9 is widely expressed during mouse embryo development. Gene Expr Patterns 5:609-617.

Kaczmarek L (2016) MMP-9 in Control of Synaptic Plasticity: a Subjective Account OM&P 2:103-111.

Larsen PH, Yong VW (2004) The expression of matrix metalloproteinase-12 by oligodendrocytes regulates their maturation and morphological differentiation. J Neurosci 24:7597-7603.

Le AP, Friedman WJ (2012) Matrix metalloproteinase-7 regulates cleavage of pro-nerve growth factor and is neuroprotective following kainic acid-induced seizures. J Neurosci 32:703-712.

Leake A, Morris CM, Whateley J (2000) Brain matrix metalloproteinase 1 levels are elevated in Alzheimer's disease. Neurosci Lett 291:201-203.

Lee EJ, Han JE, Woo MS, Shin JA, Park EM, Kang JL, Moon PG, Baek MC, Son WS, Ko YT, Choi JW, Kim HS (2014) Matrix metalloproteinase-8 plays a pivotal role in neuroinflammation by modulating TNF-alpha activation. J Immunol 193:2384-2393.

Lendeckel U, Wolke C, Bernstein HG, Keilhoff G (2015) Effects of nitric oxide synthase deficiency on a disintegrin and metalloproteinase domain-containing protein 12 expression in mouse brain samples. Molecular medicine reports 12:2253-2262.

Letronne F et al. (2016) ADAM30 Downregulates APP-Linked Defects Through Cathepsin D Activation in Alzheimer's Disease. EBioMedicine 9:278-292.

Levy C, Brooks JM, Chen J, Su J, Fox MA (2015) Cell-specific and developmental expression of lectican-cleaving proteases in mouse hippocampus and neocortex. J Comp Neurol 523:629-648.

Liao G, Wang Z, Lee E, Moreno S, Abuelnasr O, Baudry M, Bi X (2015) Enhanced expression of matrix metalloproteinase-12 contributes to Npc1 deficiency-induced axonal degeneration. Exp Neurol 269:67-74.

Liu Y, Zhang M, Hao W, Mihaljevic I, Liu X, Xie K, Walter S, Fassbender K (2013) Matrix metalloproteinase-12 contributes to neuroinflammation in the aged brain. Neurobiol Aging 34:1231-1239.

Lu DY, Yu WH, Yeh WL, Tang CH, Leung YM, Wong KL, Chen YF, Lai CH, Fu WM (2009) Hypoxia-induced matrix metalloproteinase-13 expression in astrocytes enhances permeability of brain endothelial cells. J Cell Physiol 220:163-173.

Ma F, Martinez-San Segundo P, Barcelo V, Morancho A, Gabriel-Salazar M, Giralt D, Montaner J, Rosell A (2016) Matrix metalloproteinase-13 participates in neuroprotection and neurorepair after cerebral ischemia in mice. Neurobiol Dis 91:236-246.

Malinverno M, Carta M, Epis R, Marcello E, Verpelli C, Cattabeni F, Sala C, Mulle C, Di Luca M, Gardoni F (2010) Synaptic localization and activity of ADAM10 regulate excitatory synapses through N-cadherin cleavage. J Neurosci 30:16343-16355.

Marchenko GN, Marchenko ND, Strongin AY (2003) The structure and regulation of the human and mouse matrix metalloproteinase-21 gene and protein. Biochem J 372:503-515.

Marcinkiewicz M, Seidah NG (2000) Coordinated expression of beta-amyloid precursor protein and the putative beta-secretase BACE and alpha-secretase ADAM10 in mouse and human brain. J Neurochem 75:2133-2143.

Mardinly AR, Spiegel I, Patrizi A, Centofante E, Bazinet JE, Tzeng CP, Mandel-Brehm C, Harmin DA, Adesnik H, Fagiolini M, Greenberg ME (2016) Sensory experience regulates cortical inhibition by inducing IGF1 in VIP neurons. Nature 531:371-375.

Miller JP, Holcomb J, Al-Ramahi I, de Haro M, Gafni J, Zhang N, Kim E, Sanhueza M, Torcassi C, Kwak S, Botas J, Hughes RE, Ellerby LM (2010) Matrix metalloproteinases are modifiers of huntingtin proteolysis and toxicity in Huntington's disease. Neuron 67:199-212.

Monea S, Jordan BA, Srivastava S, DeSouza S, Ziff EB (2006) Membrane localization of membrane type 5 matrix metalloproteinase by AMPA receptor binding protein and cleavage of cadherins. J Neurosci 26:2300-2312.

Moro MA, Hurtado O, Cardenas A, Romera C, Madrigal JL, Fernandez-Tome P, Leza JC, Lorenzo P, Lizasoain I (2003) Expression and function of tumour necrosis factor-alpha-converting enzyme in the central nervous system. Neuro-Signals 12:53-58.

Oh J, Woo JM, Choi E, Kim T, Cho BN, Park ZY, Kim YC, Kim DH, Cho C (2005) Molecular, biochemical, and cellular characterization of epididymal ADAMs, ADAM7 and ADAM28. Biochem Biophys Res Commun 331:1374-1383.

Ortiz RM, Karkkainen I, Huovila AP, Honkaniemi J (2005) ADAM9, ADAM10, and ADAM15 mRNA levels in the rat brain after kainic acid-induced status epilepticus. Brain research Molecular brain research 137:272-275.

Palazuelos J, Crawford HC, Klingener M, Sun B, Karelis J, Raines EW, Aguirre A (2014) TACE/ADAM17 is essential for oligodendrocyte development and CNS myelination. J Neurosci 34:11884-11896.

Py NA, Bonnet AE, Bernard A, Marchalant Y, Charrat E, Checler F, Khrestchatisky M, Baranger K, Rivera S (2014) Differential spatio-temporal regulation of MMPs in the 5xFAD mouse model of Alzheimer's disease: evidence for a pro-amyloidogenic role of MT1-MMP. Front Aging Neurosci 6:247.

Qi B, Newcomer RG, Sang QX (2009) ADAM19/adamalysin 19 structure, function, and role as a putative target in tumors and inflammatory diseases. Current pharmaceutical design 15:2336-2348.

Rao C, Foernzler D, Loftus SK, Liu S, McPherson JD, Jungers KA, Apte SS, Pavan WJ, Beier DR (2003) A defect in a novel ADAMTS family member is the cause of the belted white-spotting mutation. Development 130:4665-4672.

Rikimaru A, Komori K, Sakamoto T, Ichise H, Yoshida N, Yana I, Seiki M (2007) Establishment of an MT4-MMP-deficient mouse strain representing an efficient tracking system for MT4-MMP/MMP-17 expression in vivo using beta-galactosidase. Genes Cells 12:1091-1100.

Rossier J, Bernard A, Cabungcal JH, Perrenoud Q, Savoye A, Gallopin T, Hawrylycz M, Cuenod M, Do K, Urban A, Lein ES (2015) Cortical fast-spiking parvalbumin interneurons enwrapped in the perineuronal net express the metallopeptidases Adamts8, Adamts15 and Neprilysin. Mol Psychiatry 20:154-161.

Saftig P, Lichtenthaler SF (2015) The alpha secretase ADAM10: A metalloprotease with multiple functions in the brain. Prog Neurobiol 135:1-20.

Schlomann U, Rathke-Hartlieb S, Yamamoto S, Jockusch H, Bartsch JW (2000) Tumor necrosis factor alpha induces a metalloprotease-disintegrin, ADAM8 (CD 156): implications for neuron-glia interactions during neurodegeneration. J Neurosci 20:7964-7971.

Scott IC, Blitz IL, Pappano WN, Imamura Y, Clark TG, Steiglitz BM, Thomas CL, Maas SA, Takahara K, Cho KW, Greenspan DS (1999) Mammalian BMP-1/Tolloid-related metalloproteinases, including novel family member mammalian Tolloid-like 2, have differential enzymatic activities and distributions of expression relevant to patterning and skeletogenesis. Dev Biol 213:283-300.

Sekine-Aizawa Y, Hama E, Watanabe K, Tsubuki S, Kanai-Azuma M, Kanai Y, Arai H, Aizawa H, Iwata N, Saido TC (2001) Matrix metalloproteinase (MMP) system in brain: identification and characterization of brain-specific MMP highly expressed in cerebellum. Eur J Neurosci 13:935-948.

Sharma K, Schmitt S, Bergner CG, Tyanova S, Kannaiyan N, Manrique-Hoyos N, Kongi K, Cantuti L, Hanisch UK, Philips MA, Rossner MJ, Mann M, Simons M (2015) Cell type- and brain region-resolved mouse brain proteome. Nat Neurosci 18:1819-1831.

Somerville RP, Jungers KA, Apte SS (2004) Discovery and characterization of a novel, widely expressed metalloprotease, ADAMTS10, and its proteolytic activation. J Biol Chem 279:51208-51217.

Sterchi EE, Stocker W, Bond JS (2008) Meprins, membrane-bound and secreted astacin metalloproteinases. Mol Aspects Med 29:309-328.

Tamura G, Olson D, Miron J, Clark TG (2005) Tolloid-like 1 is negatively regulated by stress and glucocorticoids. Brain research Molecular brain research 142:81-90.

Tauchi R, Imagama S, Ohgomori T, Natori T, Shinjo R, Ishiguro N, Kadomatsu K (2012) ADAMTS-13 is produced by glial cells and upregulated after spinal cord injury. Neurosci Lett 517:1-6.

Umland SP, Garlisi CG, Shah H, Wan Y, Zou J, Devito KE, Huang WM, Gustafson EL, Ralston R (2003) Human ADAM33 messenger RNA expression profile and post-transcriptional regulation. American journal of respiratory cell and molecular biology 29:571-582.

van Horssen J, Vos CM, Admiraal L, van Haastert ES, Montagne L, van der Valk P, de Vries HE (2006) Matrix metalloproteinase-19 is highly expressed in active multiple sclerosis lesions. Neuropathol Appl Neurobiol 32:585-593.

Van Hove I, Lemmens K, Van de Velde S, Verslegers M, Moons L (2012a) Matrix metalloproteinase-3 in the central nervous system: a look on the bright side. J Neurochem 123:203-216.

Van Hove I, Verslegers M, Buyens T, Delorme N, Lemmens K, Stroobants S, Gantois I, D'Hooge R, Moons L (2012b) An aberrant cerebellar development in mice lacking matrix metalloproteinase-3. Mol Neurobiol 45:17-29.

Velasco G, Pendas AM, Fueyo A, Knauper V, Murphy G, Lopez-Otin C (1999) Cloning and characterization of human MMP-23, a new matrix metalloproteinase predominantly expressed in reproductive tissues and lacking conserved domains in other family members. J Biol Chem 274:4570-4576.

Verslegers M, Lemmens K, Van Hove I, Moons L (2013) Matrix metalloproteinase-2 and -9 as promising benefactors in development, plasticity and repair of the nervous system. Prog Neurobiol 105:60-78.

Wang J, Zhang W, Yi Z, Wang S, Li Z (2012) Identification of a thrombin cleavage site and a short form of ADAMTS-18. Biochem Biophys Res Commun 419:692-697.

Werner SR, Mescher AL, Neff AW, King MW, Chaturvedi S, Duffin KL, Harty MW, Smith RC (2007) Neural MMP-28 expression precedes myelination during development and peripheral nerve repair. Dev Dyn 236:2852-2864.

Weskamp G, Cai H, Brodie TA, Higashyama S, Manova K, Ludwig T, Blobel CP (2002) Mice lacking the metalloprotease-disintegrin MDC9 (ADAM9) have no evident major abnormalities during development or adult life. Mol Cell Biol 22:1537-1544.

Wiera G, Nowak D, van Hove I, Dziegiel P, Moons L, Mozrzymas JW (2017) Mechanisms of NMDA Receptor- and Voltage-Gated L-Type Calcium Channel-Dependent Hippocampal LTP Critically Rely on Proteolysis That Is Mediated by Distinct Metalloproteinases. J Neurosci 37:1240-1256.

Yang P, Baker KA, Hagg T (2005) A disintegrin and metalloprotease 21 (ADAM21) is associated with neurogenesis and axonal growth in developing and adult rodent CNS. J Comp Neurol 490:163-179.

Yoshinaka T, Nishii K, Yamada K, Sawada H, Nishiwaki E, Smith K, Yoshino K, Ishiguro H, Higashiyama S (2002) Identification and characterization of novel mouse and human ADAM33s with potential metalloprotease activity. Gene 282:227-236.

Yoshiyama Y, Sato H, Seiki M, Shinagawa A, Takahashi M, Yamada T (1998) Expression of the membrane-type 3 matrix metalloproteinase (MT3-MMP) in human brain tissues. Acta Neuropathol 96:347-350.
